# Concurrent Changes in Diet Quality and Physical Activity and Association With Adiposity in Adults

**DOI:** 10.1001/jamanetworkopen.2025.45232

**Published:** 2025-11-21

**Authors:** Shayan Aryannezhad, Fumiaki Imamura, Emanuella De Lucia Rolfe, Simon J. Griffin, Nicholas J. Wareham, Soren Brage, Nita G. Forouhi

**Affiliations:** 1 MRC Epidemiology Unit, University of Cambridge School of Clinical Medicine, Institute of Metabolic Science, Cambridge, United Kingdom

## Abstract

**Importance:**

Diet and physical activity (PA) are both associated with body weight, but less is understood about how long-term changes in these behaviors may be associated with changes in adiposity in the general population.

**Objective:**

To evaluate associations of concurrent changes in diet quality and PA with different body composition indices.

**Design, Setting, and Participants:**

This cohort study was conducted among participants in the UK population-based Fenland study, with repeated measurements of health behaviors and adiposity. Data collection occurred in 2005 to 2015 and 2014 to 2020, with a mean (SD) follow-up of 7.2 (2.0) years. Data were analyzed from January 2024 through April 2025.

**Exposures:**

Mediterranean diet score (MDS), a measure of adherence to the Mediterranean diet (range, 0-15 points), was assessed with a food frequency questionnaire. PA energy expenditure (PAEE), measured in kilojoules per kilogram per day, was assessed and calibrated with heart rate and movement sensing. Change variables were derived.

**Main Outcomes and Measures:**

Indices of total adiposity (weight, body mass index and body fat) and regional adiposity (waist circumference, visceral adipose tissue [VAT], and subcutaneous adipose tissue) were assessed with anthropometry and dual-energy x-ray absorptiometry. Hepatic steatosis was ascertained by abdominal ultrasonography.

**Results:**

The analysis included 7256 participants (mean [SD; range] age at recruitment, 48.8 [7.4; 29-65] years; 3748 female [51.7%]). In multivariable linear regression models after adjustment for potential confounders and baseline values, changes in both exposures were negatively associated with changes in all adiposity indices. For example, each 1-SD (1.27 points) increase in the change in MDS was negatively associated with changes in adiposity measures (change in body fat: β = −0.47 kg; 95% CI, −0.58 to −0.36 kg; change in VAT: β = −45 g; 95% CI, −55 to −35 g), and each 1-SD (19.0 kJ/kg/d) in the change in PAEE was similarly negatively associated with changes in adiposity measures (change in body fat: β = −1.40 kg; 95% CI, −1.51 to −1.26 kg; change in VAT: β = −108 g; 95% CI, −118 to −98 g) . Despite no evidence for a diet-PA interaction, simultaneous increases in MDS and PAEE were associated with a greater magnitude of decrease in adiposity, with a β of −149 g (95% CI, −187 to −111 g) for change in VAT among participants in higher joint change in MDS and change in PAEE tertiles. Associations had higher β values among individuals with overweight or obesity or who were physically inactive at baseline; for example, for each 1-SD increase in the change in PAEE, the β for change in body fat was −0.96 kg (95% CI, −1.10 to −0.81 kg) when baseline BMI was less than 25 and −1.74 kg (95% CI, −1.91 to −1.57 kg) when baseline BMI was 25 or greater (*P* for interaction < .001).

**Conclusions and Relevance:**

In this study, improved diet quality and increased PA were independently associated with weight loss or less weight gain and a healthier adiposity profile. Combining these health behaviors was associated with the greatest adiposity-related benefits.

## Key Points

**Question:**

Are there independent and joint associations of changes in diet quality and physical activity with changes in adiposity?

**Findings:**

In this cohort study of 7256 UK adults, improvements in diet quality and physical activity were independently associated with a lesser increase in total body fat and visceral fat, with the greatest benefit observed when both health behaviors improved together. The finding for visceral fat persisted after adjusting for total body fat, and no compensatory or synergistic interaction was found.

**Meaning:**

These findings suggest that improving diet quality and physical activity over time may be most effective for managing overall and central adiposity and achieving a healthier adiposity profile.

## Teaser

This cohort study investigates independent and joint associations of changes in diet quality and physical activity with body composition indices among adults in the UK.

## Introduction

Nearly 3 billion people worldwide are living with overweight or obesity.^1^ This represents a major global health concern imposing profound impacts on the incidence of cardiometabolic diseases, cancer, and premature mortality.^2^ Projections suggest that the prevalence of overweight and obesity will continue to increase unless effective preventive strategies are implemented.^1^ Research evidence for the benefits associated with diet and physical activity has largely been based on weight-loss trials among adults with overweight or obesity,^3,4^ but evidence is limited for the role of these health behaviors for the primary prevention of weight gain that often occurs gradually over years. In particular, there are research gaps for approaches that consider the complex interplay between diet and physical activity (PA) and their joint association with markers of adiposity.

Evidence of the association of diet and PA with adiposity at the population level comes mostly from prospective cohort studies that examined these 2 health behaviors separately, limiting insights into their synergistic association with weight management and metabolic health.^5,6^ Moreover, these studies used single-time assessments of health behaviors in association with later weight status, which increases measurement error and the possibility that estimates of associations may be affected by regression dilution bias. However, consistent and biologically plausible associations between lifestyle factors and long-term weight gain are observed when evaluating concurrent changes in them through repeated assessments, not through single-time assessment.^7^ This may be due to the issue of compensatory health behaviors, when a change in 1 behavior prompts a compensatory change in another in individuals living in natural settings.^8^ Small-scale, short-duration trials have reported synergism between diet quality and PA in the association with adiposity.^9^ However, this needs to be tested in larger, population-based studies and over longer follow-up periods to confirm its generalizability.

Some cohort studies^10,11,12^ have examined associations of concurrent changes in diet and PA with adiposity. However, they primarily focused on self-reported weight as the outcome, which is prone to error and bias and does not assess body fat distribution. Total body weight or body mass index (BMI; calculated as weight in kilograms divided by height in meters squared) alone do not differentiate between various adiposity components, such as visceral and subcutaneous fat or hepatic fat, which have distinct metabolic effects, but their use is currently underrepresented in the literature.^13,14^ These weight change studies^10,11,12^ also relied on self-reported PA measures, which may introduce measurement error and bias. This could be reduced through the use of objective measures, thereby improving the validity and precision of these analyses. In this study, we aimed to investigate how changes over time in overall diet quality and objectively measured PA may be associated with concurrent changes in several markers of adiposity, including body composition indices assessed using dual-energy x-ray absorptiometry (DEXA) and ultrasonography-diagnosed hepatic steatosis.

## Methods

The Fenland study is an ongoing population-based cohort study involving 12 435 adults in the UK born between 1950 and 1975 and registered with general practices in Cambridge, Ely, and Wisbech. People were excluded if they had known preexisting diabetes, were pregnant or breastfeeding, were unable to walk unaided for at least 10 minutes, had psychosis, or had a terminal illness. The study used a prospective design and collected detailed health and health behavior information from participants in phase 1 between 2005 and 2015 and phase 2 between 2014 and 2020, with a minimum interval of 4 years between the 2 phases.^15^ The Fenland study was approved by the Cambridge Local Research Ethics Committee (National Research Ethics Service Committee East of England-Cambridge Central) and was performed in agreement with the Helsinki Declaration and its former amendments. Informed consent was obtained from all participants. This study is reported following the Strengthening the Reporting of Observational Studies in Epidemiology (STROBE) reporting guideline.

### Measurements

Habitual diet was assessed using a validated 130-item food frequency questionnaire, and diet quality was evaluated with the Mediterranean diet score (MDS).^16,17,18,19^ Physical activity was measured using individually calibrated combined heart rate and movement sensors, summarized as mean daily physical activity energy expenditure (PAEE).^20,21,22,23,24,25^ Covariates included sociodemographic and behavioral characteristics assessed by questionnaire. Adiposity was assessed using anthropometry, DEXA, and waist circumference. Liver fat was measured using ultrasonography scored against standardized criteria.^26^ Full details of dietary, physical activity, covariate, and adiposity assessments are provided in the eMethods in Supplement 1.

### Statistical Analysis

Among 7831 participants who attended both baseline and follow-up visits, 193 individuals with missing food frequency questionnaire or wearable sensor data were excluded, as were 382 individuals with inadequate movement sensor time or implausible energy intake. Adequate movement sensor time was defined as a total wear time of 72 hours or more, with a sum of more than 8 hours of wear time in each day quadrant (12 am to 6 am, 6 am to 12 pm, 12 pm to 6 pm, and 6 pm to 12 am) over the wear time. Implausible energy intake was considered as that outside the ranges of 500 to 3500 kcal/d for females and 800 to 4000 kcal/d for males. Exclusions resulted in an analytical sample of 7256 participants. Depending on the availability of adiposity variables for participants, sample sizes varied by the outcome (for details of participant selection, see eFigure 1 in Supplement 1).

We used multivariable linear regression models to estimate the regression coefficient (β) and 95% CIs for associations between concurrent changes over time in mutual exposures − longitudinal within-person changes in MDS (change in MDS) and PAEE (change in PAEE) and changes in adiposity markers (change in adiposity indices). We assessed potential nonlinear associations visually using restricted cubic spline regression. Poisson regression was modeled to evaluate the association of change in MDS and change in PAEE with the risk of hepatic steatosis at the first follow-up among 4784 participants without hepatic steatosis at baseline. We adjusted primary models for potential confounders, including age, sex, baseline PAEE, baseline MDS, baseline value of the outcome adiposity marker, follow-up time, test site, education, marital status, occupation type, time-updated household income, time-updated smoking status, baseline energy intake, and change in energy intake.

We used a stepwise approach to explore whether the association was independent of total body mass or body fat (ie, specific to the localized adiposity site). This involved further adjusting models for baseline and change in height, baseline and change in BMI, and baseline and change in body fat. For hepatic steatosis, additional adjustment for baseline and change in visceral adipose tissue (VAT) was made.

To test the hypothesis that joint associations of change in MDS and change in PAEE with adiposity markers were above and beyond the summation of each individual association, we tested the change in MDS–change in PAEE interaction by including the cross-product term (change in PAEE × change in MDS) in models. To estimate joint associations additionally, categorical variables were created to represent tertiles of change in MDS and change in PAEE, categorized as decrease, stable, and increase. These categories were then cross-classified to form 9 joint trajectory groups. The stable-stable group served as the reference category in the regression model.

We conducted a series of ancillary and sensitivity analyses to assess the robustness of the main findings. These included tests for potential interactions by baseline age, sex, BMI, smoking status, MDS, and PAEE. In addition, we examined cross-sectional associations, longitudinal associations of PA and diet quality at baseline with changes in adiposity markers over the follow-up, minimally adjusted associations, and complete case analysis. We made additional adjustments for seasonality, family history of diabetes, menopause, and hormone replacement therapy in female participants. Diet quality was alternatively measured using plasma vitamin C as a biomarker of fruit and vegetable intake. Furthermore, where appropriate, we expressed adiposity markers in association with body weight, reported the incidence of overweight or obesity as a binary outcome, and expressed adiposity outcomes in SD units. Statistical analyses were conducted using Stata statistical software version 17 (StataCorp). All tests were 2-sided, and results were considered statistically significant at *P* < .05. Data were analyzed from January 2024 through April 2025.

## Results

Among 7256 participants (mean [SD; range] age at baseline, 48.8 [7.4; 29-65] years; 3748 female [51.7%]) (Table 1), a mean (SD) of 7.2 (2.0) years passed between the 2 follow-up phases. At phase 1, the mean (SD) PAEE was 54.3 (21.7) kJ/kg/d, with an increase over time (mean [SD] change in PAEE, 3.2 [19.0] kJ/kg/d). Similarly, the mean (SD) MDS at phase 1 was 7.6 (1.5) points, with a mean (SD) increase over time of 0.3 (1.3) points. The mean (SD) BMI at baseline was 26.4 (4.5), with a mean (SD) increase over time of 0.3 (2.0). Similarly, mean values for all other adiposity markers, including weight, waist circumference (WC), body fat, and VAT, increased from phase 1 to phase 2, but this was not true for subcutaneous adipose tissue (SCAT). The prevalence of hepatic steatosis was 1445 of 6229 participants with available data (23.2%) at phase 1, which increased to 1872 of 7256 participants (25.8%) at phase 2.

**Table 1.** Participant Characteristics

Characteristics and covariates	Participants, No. (%) (N = 7256)^a^		Change in variable^b^
	Phase 1 (baseline)	Phase 2 (repeated assessment)	
Age, mean (SD), y	48.8 (7.4)	56.0 (7.1)	NA
Time elapsed between phases, mean (SD), y	NA	NA	7.2 (2.0)
Sex			
Female	3748 (51.7)	NA^c^	NA
Male	3508 (48.3)	NA^c^	NA
Test site			
Cambridge	2928 (40.4)	2952 (40.7)	NA
Ely	2571 (35.4)	2586 (35.6)	NA
Wisbech	1757 (24.2)	1718 (23.7)	NA
Education level			
≤Compulsory	1206 (16.6)	NA^c^	NA
Further education	3287 (38.1)	NA^c^	NA
Higher education	2763 (45.3)	NA^c^	NA
Occupation type			
Managerial or professional	4449 (61.3)	4484 (61.8)	NA
Other job type	2527 (34.8)	2714 (37.4)	NA
Marital status			
Single	469 (6.5)	480 (6.6)	NA
Married or living as married	4840 (66.7)	6036 (83.2)	NA
Widowed, separated, or divorced	507 (7.0)	726 (10.1)	NA
Not available	745 (10.3)	14 (0.2)	NA
Annual household income, £			
<20 000	767 (10.6)	868 (12.0)	NA
20 000–40 000	2407 (33.2)	2164 (29.8)	NA
>40 000	3935 (54.2)	4045 (55.8)	NA
Smoking status:			
Never smoker	4143 (57.1)	4251 (58.6)	NA
Former smoker	2467 (34.0)	2568 (35.4)	NA
Current smoker	633 (8.7)	426 (5.9)	NA
Energy intake, mean (SD), kcal/d	1942 (562)	1847 (546)	NA
Alcohol intake, mean (SD), g/d	10.5 (13.4)	9.3 (12.0)	NA
**Exposures and outcomes**			
PAEE, mean (SD), kJ/kg/d	54.3 (21.7)	57.5 (22.7)	3.2 (19.0)
MDS, mean (SD), points	7.6 (1.5)	8.0 (1.5)	0.3 (1.3)
Weight, mean (SD), kg	77.4 (15.7)	77.9 (16.1)	0.6 (5.7)
BMI, mean (SD)	26.4 (4.5)	26.7 (4.7)	0.3 (2.0)
WC, mean (SD), cm	90.1 (13.0)	92.1 (13.5)	2.1 (6.3)
Body fat, mean (SD), kg	25.6 (8.8)	26.5 (9.3)	0.9 (4.6)
Body fat, percentage of total body weight	32.8 (7.5)	33.8 (7.9)	1.0 (3.5)
VAT, mean (SD), g	945 (763)	1078 (851)	145 (400)
VAT proportion of total body weight, mean (SD), %	1.1 (0.8)	1.3 (0.8)	0.2 (0.4)
SCAT, mean (SD), g	1320 (651)	1310 (621)	−1 (343)
SCAT proportion of total body weight, mean (SD), %	1.7 (0.7)	1.7 (0.7)	0 (0.3)
Hepatic steatosis	1445 (23.2)	1872 (25.8)	2.6

Abbreviations: BMI, body mass index (calculated as weight in kilograms divided by height in meters squared); MDS, Mediterranean diet score; NA, not applicable; PAEE, physical activity energy expenditure; SCAT, subcutaneous adipose tissue; VAT, visceral adipose tissue; WC, waist circumference.

^a^ There were 7256 participants with data available at both study phases.

^b^ Changes over time in variables were calculated as phase 2 − phase 1.

^c^ For sex and education levels, phase 2 status was identical to that of phase 1.

In multivariable regression models adjusted for potential confounders, each 1-SD (19.0 kJ/kg/d) increase in change in PAEE was negatively associated with changes in all adiposity markers (change in body fat: β = −1.40 kg; 95% CI, −1.51 to −1.26 kg; change in VAT: β = −108 g; 95% CI, −118 to −98 g) and each 1-SD (1.27 points) increase in change in MDS was also negatively associated with changes in all adiposity marker (change in body fat: β = −0.47 kg; 95% CI, −0.58 to −0.36 kg; change in VAT: β = −45 g; 95% CI, −55 to −35 g) (Table 2). The direction of the associations was similar for change in PAEE and change in MDS, but the magnitude of the β in these associations was greater for change in PAEE compared with change in MDS. There was no evidence of interaction between change in PAEE and change in MDS for any outcome (eTable 1 in Supplement 1).

**Table 2.** Associations of Concurrent Changes in PA and Diet With Adiposity Hepatic Steatosis

Outcome	Point estimate (95% CI)^a^	
	Per change in PAEE^b^	Per change in MDS^c^
Change in adiposity markers, β coefficient^d^		
Weight, kg	−1.59 (−1.73 to −1.45)	−0.52 (−0.66 to −0.38)
BMI	−0.54 (−0.59 to −0.49)	−0.19 (−0.24 to −0.15)
WC, cm	−1.7 (−1.8 to −1.5)	−0.7 (−0.8 to −0.5)
Body fat, kg	−1.40 (−1.51 to −1.26)	−0.47 (−0.58 to −0.36)
SCAT, g	−75 (−83 to −67)	−28 (−36 to −20)
VAT, g	−108 (−118 to −98)	−45 (−55 to −35)
Hepatic steatosis, IRR^e^	0.80 (0.74 to 0.87)	0.89 (0.82 to 0.96)

Abbreviations: BMI, body mass index (calculated as weight in kilograms divided by height in meters squared); change in MDS, changes in Mediterranean diet score over time; change in PAEE, changes in physical activity energy expenditure over time; IRR, incidence rate ratio; SCAT, subcutaneous adipose tissue; VAT, visceral adipose tissue; WC, waist circumference.

^a^ Associations are shown of changes over time in factors with adiposity markers and incidence of hepatic steatosis.

^b^ Per 1-SD increase in change in PAEE (equivalent to 19.0 kJ/kg/d).

^c^ Per 1-SD increase in change in MDS (equivalent to 1.27 points).

^d^ Changes over time in markers of adiposity (value at phase 2 − value at phase 1) in multivariable linear regression with adjustment for mutual exposures and confounders, including age, sex, baseline PAEE, baseline MDS, baseline value of the outcome adiposity marker, follow-up time, test site, education, marital status, occupation type, time-updated annual household income, time-updated smoking status, energy intake at baseline, and change in energy intake.

^e^ Incidence of ultrasonography-diagnosed hepatic steatosis at phase 2 in participants without hepatic steatosis at phase 1 in Poisson regression, with models adjusted as described for the multivariable linear regression.

A series of stepwise adjustments for other adiposity markers was conducted to evaluate whether observed associations were independent or mediated through changes in other adiposity markers (Table 3). Adjustment for BMI attenuated observed associations for change in WC, change in body fat, and change in VAT, but those associations remained. For change in SCAT, adjusting for BMI nullified associations, and further adjustment for body fat showed the association of change in PAEE (β = 7.16 g; 95% CI, 1.68 to 12.64 g per 1-SD in change in PAEE) to be positive while there was no association for change in MDS (β = 0.28 g; 95% CI, −4.89 to 5.45 g per 1-SD change in MDS). For hepatic steatosis, there were no longer associations after further adjustment for BMI, body fat, or VAT.

**Table 3.** Associations of PA and Diet in Multivariable Linear Regression With Adjustments

Outcome	Point estimate coefficient (95%CI)^a^	
	Per change in PAEE^b^	Per change in MDS^c^
**Adiposity marker, β coefficient^d^**		
Change in weight, kg		
+Adjusted for baseline height	−1.61 (−1.76 to −1.47)	−0.53 (−0.67 to −0.40)
+Adjusted for change in height	−1.58 (−1.72 to −1.44)	−0.53 (−0.67 to −0.39)
Change in WC, cm		
+Adjusted for baseline height	−1.68 (−1.83 to −1.52)	−0.68 (−0.83 to −0.52)
+Adjusted for change in height	−1.66 (−1.82 to −1.50)	−0.68 (−0.83 to −0.52)
+Adjusted for baseline BMI	−1.59 (−1.74 to −1.44)	−0.67 (−0.82 to −0.52)
+Adjusted for change in BMI	−0.22 (−0.31 to −0.13)	−0.20 (−0.29 to −0.11)
+Adjusted for baseline body fat	−0.16 (−0.25 to −0.07)	−0.17 (−0.26 to −0.08)
+Adjusted for change in body fat	−0.08 (−0.18 to 0.01)	−0.15 (−0.24 to −0.07)
Change in body fat, kg		
+Adjusted for baseline height	−1.41 (−1.52 to −1.29)	−0.48 (−0.59 to −0.36)
+Adjusted for change in height	−1.38 (−1.50 to −1.27)	−0.47 (−0.58 to −0.36)
+Adjusted for baseline BMI	−1.39 (−1.51 to −1.28)	−0.47 (−0.58 to −0.36)
+Adjusted for change I BMI	−0.18 (−0.22 to −0.14)	−0.04 (−0.08 to 0.00)
Change in SCAT, g		
+Adjusted for baseline height	−75.22 (−83.59 to −66.84)	−28.30 (−36.54 to −20.05)
+Adjusted for change in height	−74.48 (−82.86 to −66.09)	−28.22 (−36.46 to −19.98)
+Adjusted for baseline BMI	−72.81 (−81.21 to −64.42)	−27.12 (−35.36 to −18.88)
+Adjusted for change in BMI	−3.99 (−9.84 to 1.86)	−2.47 (−8.05 to 3.11)
+Adjusted for baseline body fat	−2.99 (−8.87 to 2.89)	−2.08 (−7.66 to 3.50)
+Adjusted for change in body fat	7.16 (1.68 to 12.64)	0.28 (−4.89 to 5.45)
Change in VAT, g		
+Adjusted for baseline height	−107.81 (−118.07 to −97.55)	−45.35 (−55.47 to −35.22)
+Adjusted for change in height	−105.12 (−115.31 to −94.93)	−45.04 (−55.09 to −35.00)
+Adjusted for baseline BMI	−103.57 (−113.78 to −93.36)	−44.34 (−54.38 to −34.30)
+Adjusted for change in BMI	−21.45 (−28.68 to −14.23)	−15.76 (−22.66 to −8.86)
+Adjusted for baseline body fat	−21.05 (−28.30 to −13.79)	−15.66 (−22.56 to −8.76)
+Adjusted for change in body fat	−8.79 (−15.54 to −2.03)	−12.68 (−19.08 to −6.29)
**Hepatic steatosis, IRR^e^**		
+Adjusted for baseline BMI	0.86 (0.79 to 0.93)	0.92 (0.85 to 0.99)
+Adjusted for change in BMI	0.96 (0.89 to 1.05)	0.96 (0.89 to 1.04)
+Adjusted for baseline body fat	0.99 (0.91 to 1.08)	0.96 (0.89 to 1.04)
+Adjusted for change in body fat	1.00 (0.92 to 1.09)	0.97 (0.89 to 1.04)
+Adjusted for baseline VAT	0.99 (0.91 to 1.08)	0.96 (0.89 to 1.04)
+Adjusted for change in VAT	1.00 (0.92 to 1.09)	0.97 (0.90 to 1.05)

Abbreviations: BMI, body mass index (calculated as weight in kilograms divided by height in meters squared); change in MDS, changes in Mediterranean diet score over time; change in PAEE, changes in physical activity energy expenditure over time; IRR, incidence rate ratio; SCAT, subcutaneous adipose tissue; VAT, visceral adipose tissue; WC, waist circumference.

^a^ Associations are shown of concurrent changes over time in factors with adiposity markers and incidence of hepatic steatosis with adjustment for confounders and other adiposity markers. Each subsequent subrow with plus sign (+) indicates that the adjustment is in addition to adjustments in previous subrows.

^b^ Per 1-SD increase in change in PAEE (equivalent to 19.0 kJ/kg/d).

^c^ Per 1-SD increase in change in MDS (equivalent to 1.27 points).

^d^ Changes over time in markers of adiposity (value at phase 2 − value at phase 1). Multivariable linear regression was adjusted for mutual exposures and confounders, including age, sex, baseline PAEE, baseline MDS, baseline value of the outcome adiposity marker, follow-up time, test site, education, marital status, occupation type, time-updated household annual income, time-updated smoking status, energy intake at baseline, and change in energy intake, plus a stepwise addition of other adiposity markers into the model.

^e^ Incidence of ultrasonography-diagnosed hepatic steatosis at phase 2 in participants without hepatic steatosis at phase 1. Poisson regression models were adjusted as described for the multivariable linear regression.

**Figure 1.**
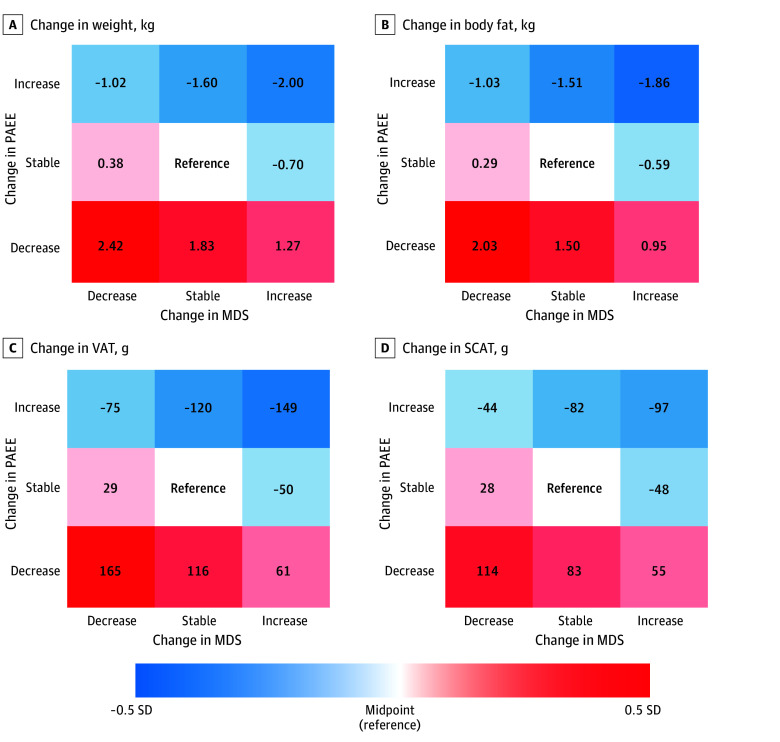
Joint Associations of Concurrent Changes Over Time in Physical Activity and Diet Quality With Adiposity Blue to red colors indicate −0.5 SD to 0.5 SD in the change in the adiposity marker compared with the reference. Change in MDS indicates changes in Mediterranean diet score over time; change in PAEE, changes in physical activity energy expenditure over time; SCAT, subcutaneous adipose tissue; VAT, visceral adipose tissue.

Figure 1 shows joint associations of changes in health behaviors with changes in adiposity markers on SD scales, allowing for a comparison across different adiposity markers. In joint association analyses, increases in change in MDS or change PAEE, when not in opposite directions, were negatively associated with adiposity changes across all markers compared with relatively stable change in MDS and change in PAEE. In contrast, decreases were positively associated with adiposity changes across all markers (Figure 1; eFigure 2 and eTable 2 in Supplement 1). Similarly, individuals who increased diet quality and activity concurrently experienced the most adiposity loss, while those who reduced both exposures had the greatest adiposity gain during the follow-up. For example, simultaneous increases in MDS and PAEE were associated with a greater magnitude of decrease in adiposity (β = −149 g; 95% CI, −187 to −111 g for change in VAT among participants in higher joint change in MDS and change in PAEE tertiles). Participants whose diet and activity behaviors changed in opposite directions experienced associations with adiposity changes that had modest β values. In the decrease-decrease trajectory group, the association with the largest positive β among body fat measures was for change in body fat (β = 2.03 kg; 95% CI, 1.60 to 2.47 kg, which corresponds to 0.44 SD of the mean[SD] change in body fat over follow-up of 0.91 [4.56] kg) and the smallest for change in SCAT (β = 114 g; 95% CI, 82 to 146 g, which corresponds to 0.33 SD of the mean [SD] change in SCAT over follow-up of −1 g [343] g). In the increase-increase group, the association with the largest negative β among body fat measures was for change in body fat (β = −1.86 kg; 95% CI, −2.28 to −1.44 kg, which corresponds to 0.40 SD of the mean [SD] change in body fat over follow-up) and the smallest for change in SCAT (β = −97 g; 95% CI, −128 to −66 g, which corresponds to 0.28 SD of the change in SCAT over follow-up).

**Figure 2.**
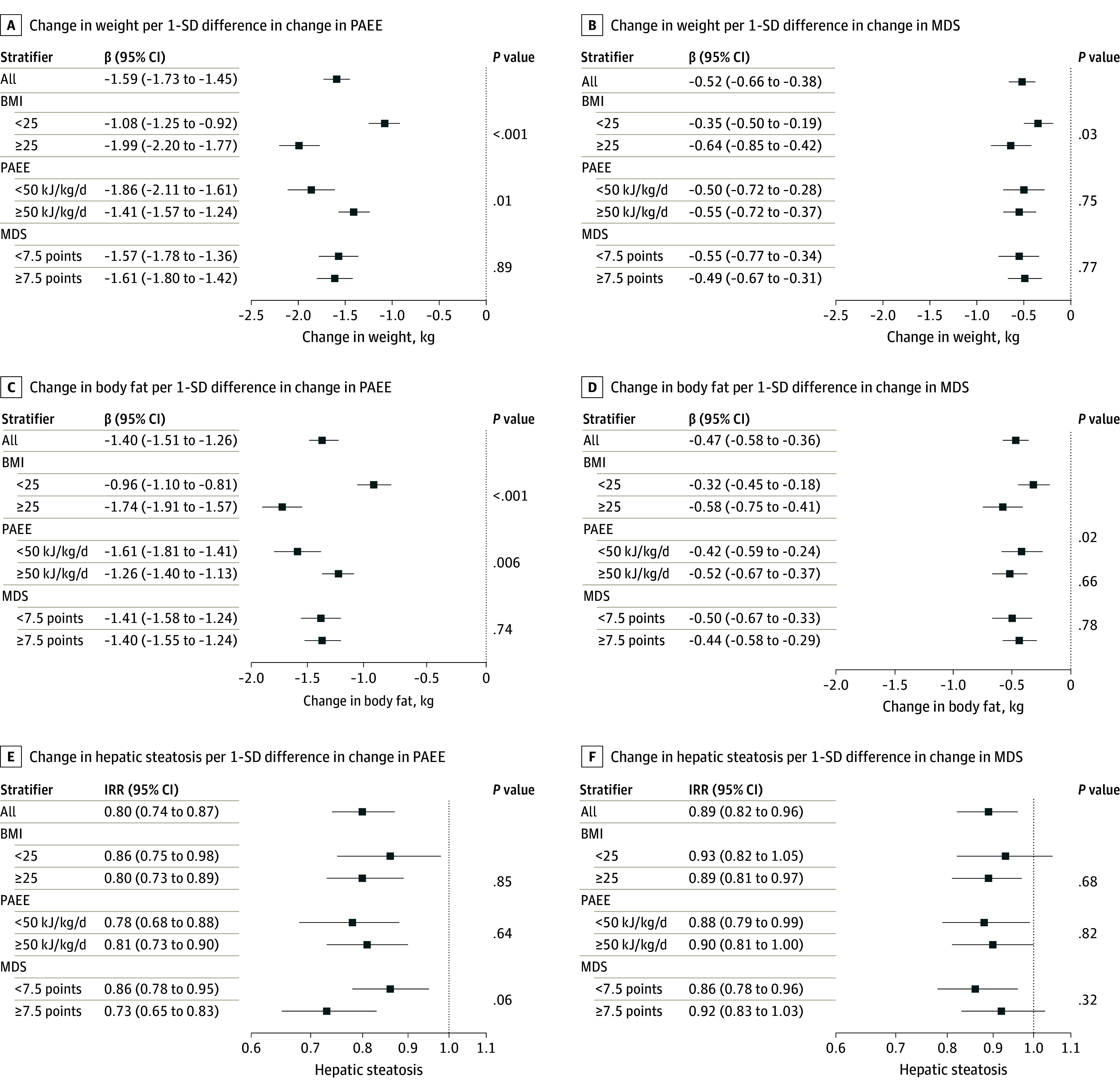
Stratified Results Associations are shown of concurrent changes over time in physical activity and diet quality with adiposity markers in different strata of baseline body mass index (BMI; calculated as weight in kilograms divided by height in meters squared), physical activity energy expenditure (PAEE), and Mediterranean diet score (MDS). Results are shown as β coefficients and 95% CIs from multivariable linear regression analyses and incidence rate ratios (IRRs) and 95% CIs from Poisson regression. Change in PAEE indicates changes in PAEE over time; change in MDS, changes in MDS over time. *P* values are for subgroup interaction.

For change in MDS and change in PAEE, interactions by baseline BMI were observed for changes in weight, BMI, body fat, and VAT (Figure 2; eTable 3 and eTable 4 in Supplement 1). There was no evidence for interaction that indicated heterogeneous directions in an association between strata. For change in body fat, for example, per 1-SD difference in change in PAEE the β was −0.96 kg (95% CI, −1.10 to −0.81 kg) when baseline BMI was 25 and −1.74 kg (95% CI, −1.91 to −1.57 kg) when baseline BMI was 25 (*P* for interaction < .001). For change in PAEE, interactions were observed depending on baseline PAEE, with a greater magnitude of associations in individuals with lower baseline PAEE. Change in MDS showed no evidence for any interaction based on baseline MDS.

Findings were consistent across sensitivity analyses of the main approach, including minimally adjusted models (eTable 5 in Supplement 1), complete case analysis (eTable 6 in Supplement 1), and models additionally adjusted for seasonality (eTable 7 in Supplement 1), as well as family history of diabetes, menopause, and hormone-replacement therapy in female participants (eTable 8 in Supplement 1). No interactions were observed by baseline menopause or hormone-replacement therapy status in female participants. In another sensitivity analysis, diet quality measured via plasma vitamin C yielded similar results to those based on MDS (eTable 9 in Supplement 1). Ancillary analyses supported the main approach, including cross-sectional associations (eTable 10 in Supplement 1) and models assessing associations between phase 1 exposures and changes in adiposity (eTable 11 in Supplement 1), when adiposity was expressed as a proportion of total body weight (eTable 12 in Supplement 1), in SD units (eTable 13 in Supplement 1), or as the incidence of overweight or obesity (eTable 14 in Supplement 1). Spline regression showed linear associations of change in PAEE and change in MDS with all adiposity outcomes (eFigures 3 and 4 in Supplement 1).

## Discussion

Using repeated measure data from the Fenland study, this cohort study found that increases in diet quality and physical activity over time were independently associated with concurrent decreases or less gain in weight, overall fat mass, and regional fat and a lower incidence of hepatic steatosis. Simultaneous improvements in both behaviors were associated with the greatest adiposity loss, while improvement in only 1 behavior was associated with relatively modest changes in body composition. Even after adjusting for BMI (a crude measure of total mass loss of all types), associations of change in MDS and change in PAEE with adiposity markers known to be metabolically unhealthy (ie, change in WC, change in body fat, and change in VAT) remained, although attenuated, as expected due to mediation through overall weight change. In contrast, after adjustment for BMI, associations with change in SCAT, a more metabolically healthy adiposity marker,^27^ were not present, and after further adjustment for body fat, the association of improving health behaviors with change in SCAT became positive. This suggests that the potential beneficial outcomes associated with changes in diet quality and physical activity may be strongly associated with reductions in unhealthy fat depots, such as VAT and central adiposity, and if anything, positively associated with SCAT when accounting for the role of other adiposity markers. Therefore, targeting improvements in diet and physical activity may be not only associated with weight loss, but also particularly effective in associations with reduced metabolically harmful fat and an overall healthier adiposity profile, which could have important implications for long-term health outcomes.

Furthermore, assessing comparative changes in different adiposity markers based on SD units showed that various body composition indices had different outcomes in association with changes in diet and activity. Total body fat and VAT showed the greatest degree of alternations over time, while SCAT exhibited smaller changes. This may be due to structural and functional differences between different adipose depots. VAT is more metabolically active and prone to mobilization during energy deficits because it is more vascular and innervated and contains a larger number of inflammatory and immune cells compared with SCAT, which is less insulin resistant and serves as a more stable fat deposit.^28^ Previous reviews suggested that VAT loss generally mirrors overall fat loss^29,30^; however, we found that combined diet and activity improvements may be associated with different outcomes for VAT and SCAT.

Our finding of an interaction between both health behaviors and baseline adiposity suggests that the potential benefits associated with improving diet quality and increasing physical activity are greater in individuals with overweight or obesity than those with reference range weight. Similar to our findings, results from other cohorts demonstrated stronger associations between changes in lifestyle habits and weight change in adults with overweight.^10,31,32^ For change in PAEE, an interaction was observed with baseline physical activity levels, indicating that individuals who were less active at baseline experienced potential benefits associated with increases in physical activity over time. These findings could be useful in guiding more personalized health advice, with potential tailoring of health behavior interventions based on individual characteristics.

Consistent with our findings, a study in the US^33^ reported that each 1-SD increase in a diet quality index was inversely associated with changes in BMI (−0.39), WC (−0.90 cm), and weight (−1.14 kg). Compared with our findings, their larger effect sizes may be due to the longer follow-up (20 years vs 7.2 years) and greater weight gain in their population (15.8 kg vs 0.6 kg). Thus, their results reflect associations between diet and decreased adiposity gain, while we observed associations with adiposity loss. Also in line with our findings, previous studies have reported that increasing activity levels over time are associated with less weight and WC gain.^34,35,36^ However, a direct comparison of their results to ours is challenging due to the use of different PA exposure units. Additionally, their findings are limited by the lack of adjustment for diet quality as a confounding factor, time-varying confounders, and the reliance on self-reported, subjective PA measures unlike our objective assessment of PA. Results from 3 US cohorts^10,11,12^ that have examined the joint associations of 4-year changes in diet quality and PA with weight change demonstrated that concurrent improvements in both behaviors were associated with the least amount of weight gain, without any interaction between diet and PA, which is consistent with our findings. We have advanced these findings by incorporating continuous measures of PA and diet quality in our models, which increases the precision of our estimates; using objectively measured PA; and using detailed DEXA-measured adiposity indices instead of relying on self-reported weight. These approaches collectively allow for a more accurate and comprehensive assessment of the association between health behavior changes and various body composition indices.

### Strengths and Limitations

Strengths of this study include the use of objective and validated assessment methods for health behavior exposures, detailed DEXA-measured assessments of adiposity, and repeated measurements of exposures and outcomes, which allowed for a change-on-change analysis over time in a large sample, enhancing the accuracy and robustness of our findings. Our study also has some limitations, including its observational design, which means we cannot establish causality; the potential lack of generalizability to other race and ethnicity groups due to the predominance of White participants in our study; and the inevitable presence of measurement errors and residual confounding that is common in observational studies, although we adjusted for a comprehensive range of potential confounding factors. Additionally, lean mass measures were not included in outcomes of this study but would be relevant to more fully characterizing body composition changes.

## Conclusions

Findings from this cohort study suggest that increasing diet quality and PA was associated with not only weight loss, but also a healthier overall adiposity profile, as demonstrated by improvements in different measures of fat distribution. Although improvements in either behavior alone were associated with lower adiposity over time, addressing both behaviors simultaneously could be associated with a substantial increase in the magnitude of potential adiposity benefits. These findings emphasize the importance of targeting both health behaviors to reduce unhealthy fat depots and promote better metabolic health.

## Notes

Supplement 1. eMethods. Full details of dietary, physical activity, covariate, and adiposity assessments eFigure 1. Flow diagram illustrating selection of the analytical sample in Fenland study eTable 1. Tests of interaction between concurrent changes over time in physical activity and diet quality in relation to adiposity markers in the Fenland study eTable 2. Joint associations of concurrent changes over time in physical activity and diet quality with adiposity markers in the Fenland study eTable 3. Associations of concurrent changes over time in physical activity with adiposity markers in the Fenland study (from phase 1 to phase 2) per 1-SD difference in PAEE in different strata of baseline sex, age, BMI, smoking, PAEE, and MDS eTable 4. Associations of concurrent changes over time in diet quality with adiposity markers in the Fenland study (from phase 1 to phase 2) per 1-SD difference in MDS in different strata of baseline sex, age, BMI, smoking, PAEE, and MDS eFigure 2. Joint associations of concurrent changes over time in physical activity and diet quality with BMI and WC in the Fenland study eTable 5. Minimally adjusted associations of concurrent changes over time in physical activity and diet quality with adiposity markers (from phase 1 to hase 2) in the Fenland study eTable 6. Associations of concurrent changes over time in physical activity and diet quality with adiposity markers (from phase 1 to phase 2) in the Fenland study, complete case analysis eTable 7. Associations of concurrent changes over time in physical activity and diet quality with adiposity markers (from phase 1 to phase 2) in the Fenland study, additionally adjusted for seasonality eTable 8. Associations of concurrent changes over time in physical activity and diet quality with adiposity markers in the Fenland study, additionally adjusted for family history of diabetes, menopause, and hormone-replacement therapy in females eTable 9. Associations of concurrent changes over time in physical activity and diet quality (measured via plasma vitamin C, a biomarker of fruit and vegetable intake) with adiposity markers (from phase 1 to phase 2) in the Fenland study eTable 10. Cross-sectional associations of physical activity and diet quality with adiposity markers in the Fenland study at phase 1 eTable 11. Longitudinal associations of baseline physical activity and diet quality (phase 1) with subsequent changes in adiposity markers (from phase 1 to phase 2) in the Fenland study eTable 12. Associations of concurrent changes over time in physical activity and diet quality with adiposity markers relative to body weight in the Fenland study (from phase 1 to phase 2) eTable 13. Associations of concurrent changes over time in physical activity and diet quality with adiposity markers in the Fenland study, expressed in SD units eTable 14. Longitudinal associations of baseline physical activity and diet quality (at phase 1) and changes over time in them (from phase 1 to phase 2) with incidence of overweight or obesity (from phase 1 to phase 2) in the Fenland study eFigure 3. Dose-response association between changes over time in PA ( PAEE) and changes in adiposity markers using restricted cubic spline regression eFigure 4. Dose-response association between changes over time in diet quality (MDS) and changes in adiposity markers using restricted cubic spline regression eReferences.

Supplement 2.
Data Sharing Statement
