# Supplementary material for: Concurrent Changes in Diet Quality and Physical Activity and Association With Adiposity in Adults
Source: JAMA Netw Open. 2025 Nov 21;8(11):e2545232. doi: 10.1001/jamanetworkopen.2025.45232 (PMC12639474; doi:10.1001/jamanetworkopen.2025.45232)
Supplement: Supplement 1. — eMethods. Full details of dietary, physical activity, covariate, and adiposity assessments eFigure 1. Flow diagram illustrating selection of the analytical sample in Fenland study eTable 1. Tests of interaction between concurrent changes over time in physical activity and diet quality in relation to adiposity markers in the Fenland study eTable 2. Joint associations of concurrent changes over time in physical activity and diet quality with adiposity markers in the Fenland study eTable 3. Associations of concurrent changes over time in physical activity with adiposity markers in the Fenland study (from phase 1 to phase 2) per 1-SD difference in PAEE in different strata of baseline sex, age, BMI, smoking, PAEE, and MDS eTable 4. Associations of concurrent changes over time in diet quality with adiposity markers in the Fenland study (from phase 1 to phase 2) per 1-SD difference in MDS in different strata of baseline sex, age, BMI, smoking, PAEE, and MDS eFigure 2. Joint associations of concurrent changes over time in physical activity and diet quality with BMI and WC in the Fenland study eTable 5. Minimally adjusted associations of concurrent changes over time in physical activity and diet quality with adiposity markers (from phase 1 to hase 2) in the Fenland study eTable 6. Associations of concurrent changes over time in physical activity and diet quality with adiposity markers (from phase 1 to phase 2) in the Fenland study, complete case analysis eTable 7. Associations of concurrent changes over time in physical activity and diet quality with adiposity markers (from phase 1 to phase 2) in the Fenland study, additionally adjusted for seasonality eTable 8. Associations of concurrent changes over time in physical activity and diet quality with adiposity markers in the Fenland study, additionally adjusted for family history of diabetes, menopause, and hormone-replacement therapy in females eTable 9. Associations of concurrent changes over time in physical activity and di [file jamanetwopen-e2545232-s001.pdf]

Supplemental Online Content

Aryannezhad S, Imamura F, De Lucia Rolfe E, et al. Concurrent Changes in Diet Quality and Physical Activity and Association With Adiposity in Adults. *JAMA Netw Open*. 2025;8(11):e2545232. doi:10.1001/jamanetworkopen.2025.45232

eMethods. Full details of dietary, physical activity, covariate, and adiposity assessments
eFigure 1. Flow diagram illustrating selection of the analytical sample in Fenland study
eTable 1. Tests of interaction between concurrent changes over time in physical activity and diet quality in relation to adiposity markers in the Fenland study
eTable 2. Joint associations of concurrent changes over time in physical activity and diet quality with adiposity markers in the Fenland study
eTable 3. Associations of concurrent changes over time in physical activity with adiposity markers in the Fenland study (from phase 1 to phase 2) per 1-SD difference in PAEE in different strata of baseline sex, age, BMI, smoking, PAEE, and MDS
eTable 4. Associations of concurrent changes over time in diet quality with adiposity markers in the Fenland study (from phase 1 to phase 2) per 1-SD difference in MDS in different strata of baseline sex, age, BMI, smoking, PAEE, and MDS
eFigure 2. Joint associations of concurrent changes over time in physical activity and diet quality with BMI and WC in the Fenland study
eTable 5. Minimally adjusted associations of concurrent changes over time in physical activity and diet quality with adiposity markers (from phase 1 to hase 2) in the Fenland study
eTable 6. Associations of concurrent changes over time in physical activity and diet quality with adiposity markers (from phase 1 to phase 2) in the Fenland study, complete case analysis
eTable 7. Associations of concurrent changes over time in physical activity and diet quality with adiposity markers (from phase 1 to phase 2) in the Fenland study, additionally adjusted for seasonality
eTable 8. Associations of concurrent changes over time in physical activity and diet quality with adiposity markers in the Fenland study, additionally adjusted for family history of diabetes, menopause, and hormone-replacement therapy in females
eTable 9. Associations of concurrent changes over time in physical activity and diet quality (measured via plasma vitamin C, a biomarker of fruit and vegetable intake) with adiposity markers (from phase 1 to phase 2) in the Fenland study
eTable 10. Cross-sectional associations of physical activity and diet quality with adiposity markers in the Fenland study at phase 1
eTable 11. Longitudinal associations of baseline physical activity and diet quality (phase 1) with subsequent changes in adiposity markers (from phase 1 to phase 2) in the Fenland study
eTable 12. Associations of concurrent changes over time in physical activity and diet quality with adiposity markers relative to body weight in the Fenland study (from phase 1 to phase 2)
eTable 13. Associations of concurrent changes over time in physical activity and diet quality with adiposity markers in the Fenland study, expressed in SD units
eTable 14. Longitudinal associations of baseline physical activity and diet quality (at phase 1) and changes over time in them (from phase 1 to phase 2) with incidence of overweight or obesity (from phase 1 to phase 2) in the Fenland study
eFigure 3. Dose-response association between changes over time in PA (PAEE) and changes in adiposity markers using restricted cubic spline regression
eFigure 4. Dose-response association between changes over time in diet quality (MDS) and changes in adiposity markers using restricted cubic spline regression
eReferences.

This supplemental material has been provided by the authors to give readers additional information about their work.

© 2025 Aryannezhad S et al. *JAMA Network Open*.

eMethods - Full details of dietary, physical activity, covariate, and adiposity assessments

Diet

Habitual diet was assessed using a 130-item semi-quantitative food frequency questionnaire (EPIC-Norfolk FFQ) asking about participants' average intake of the food items over the previous year. The validity of the FFQ for measuring nutrient, food items, and energy intake was previously evaluated using another dietary assessment tool (24-hour recall), a 16-day weighed dietary record (considered the 'gold standard' method), and biomarkers (an objective dietary assessment method) (1). To assess overall diet quality, adherence to a predefined healthy dietary pattern was evaluated using the pyramid-based Mediterranean Diet Score (MDS), first described by the Mediterranean Diet Foundation (2). We previously confirmed the relevance and applicability of the MDS in a non-Mediterranean setting, in a cross-sectional (3) and a prospective study (4) in the UK. Higher MDS scores were inversely associated with incidence of cardiovascular disease and mortality in the EPIC-Norfolk study (4) and with hepatic steatosis in the Fenland study population (3). The scoring criteria for the MDS have been detailed previously (4). To summarise, the MDS is a continuous score ranging from 0 to 15 points, reflecting how closely reported dietary intake aligns with the dietary pyramid recommendations. This score is based on 15 dietary components: vegetables, legumes, fruits, nuts, cereals, dairy, fish, red meat, processed meat, white meat, eggs, potatoes, sweets, alcohol, and olive oil.

Physical activity

Participants were asked to wear a combined heart rate and uniaxial movement sensor (Actiheart, CamNtech, Papworth, UK) which was attached to their chest via standard ECG electrodes, collecting data at 1-min resolution for 6 days (5). This device was individually calibrated using a submaximal treadmill test performed prior to wearing the device (6). Upon the return of the device, heart rate was pre-processed to remove sensor noise (7), and a branched equation model was used to calculate instantaneous PAEE (J/kg/min), utilising combination of heart rate, accelerometry and treadmill-derived calibration equation parameters (8). Then, average daily PAEE (kJ/kg/day) was summarised whilst accounting for periods when the sensor was not worn. This method of estimating PAEE in free-living individuals has been validated against doubly labelled water in UK men and women, with no apparent mean bias (9). We have previously shown PAEE from this method to be cross-sectionally inversely associated with body fatness in the Fenland study population (10). For the present analyses of change, we used the highest available individual calibration level available for both phases.

Covariates

Socio-demographic and health behaviour data were collected through self-report at baseline and repeated assessment, including age, sex, marital status (single, married, widowed/separated/divorced), education (compulsory, further education, higher education), annual household income (<£20,000, £20,000–£40,000, >£40,000), occupation type (managerial/professional, other job types), smoking status (never, former, current) and location (Cambridge, Ely, Wisbech). Participants who had missing covariate data were kept in the main

© 2025 Aryannezhad S et al. *JAMA Network Open*.

analyses by coding a missing indicator category. Other covariates included seasonality of assessment, expressed as two cosinor parameters for both baseline and follow-up dates.

Adiposity

Markers of adiposity were collected by trained personnel following standard operating procedures. Weight was measured with a calibrated electronic scale (TANIA model BC-418 MA; Tanita, Tokyo, Japan), and height was assessed with a calibrated stadiometer (SECA 240; Seca, Birmingham, United Kingdom). Waist circumference (WC) was measured at the midpoint between the lowest rib margin and the iliac crest to the nearest 0.1 cm using a non-stretchable, fiberglass insertion tape. Body mass index (BMI) was calculated by dividing weight in kilograms by height in metres squared. Adiposity was determined by DEXA using a Lunar Prodigy advanced fan beam and iDEXA scanners (GE Healthcare, Hatfield, UK) using the encore v14.10.022 and CoreScan® software (GE Healthcare, Hatfield UK), determining body composition indices related to total adiposity (body fat [BF], percentage of body fat relative to total body weight [%BF]) and regional adiposity (visceral adipose tissue [VAT], percentage of VAT relative to total body weight [%VAT], subcutaneous adipose tissue [SCAT], and percentage of SCAT relative to total body weight [%SCAT]). Liver fat was assessed using ultrasonography (LOGIQ Book XP ultrasound system; GE Healthcare, Bedford, UK) and images scored according to standardised criteria by two trained operators who were unaware of other study measures (3). Scoring criteria were increased echotexture of the liver parenchyma, decreased visualisation of the intra-hepatic vasculature, and attenuation of the ultrasound beam, each scored on a 4-point scale, summing to a liver fat score (range: 3–12). The ultrasound assessment of hepatic steatosis has previously been validated against magnetic resonance spectroscopy with a sensitivity of 96% and specificity of 94% (11).

© 2025 Aryannezhad S et al. *JAMA Network Open*.

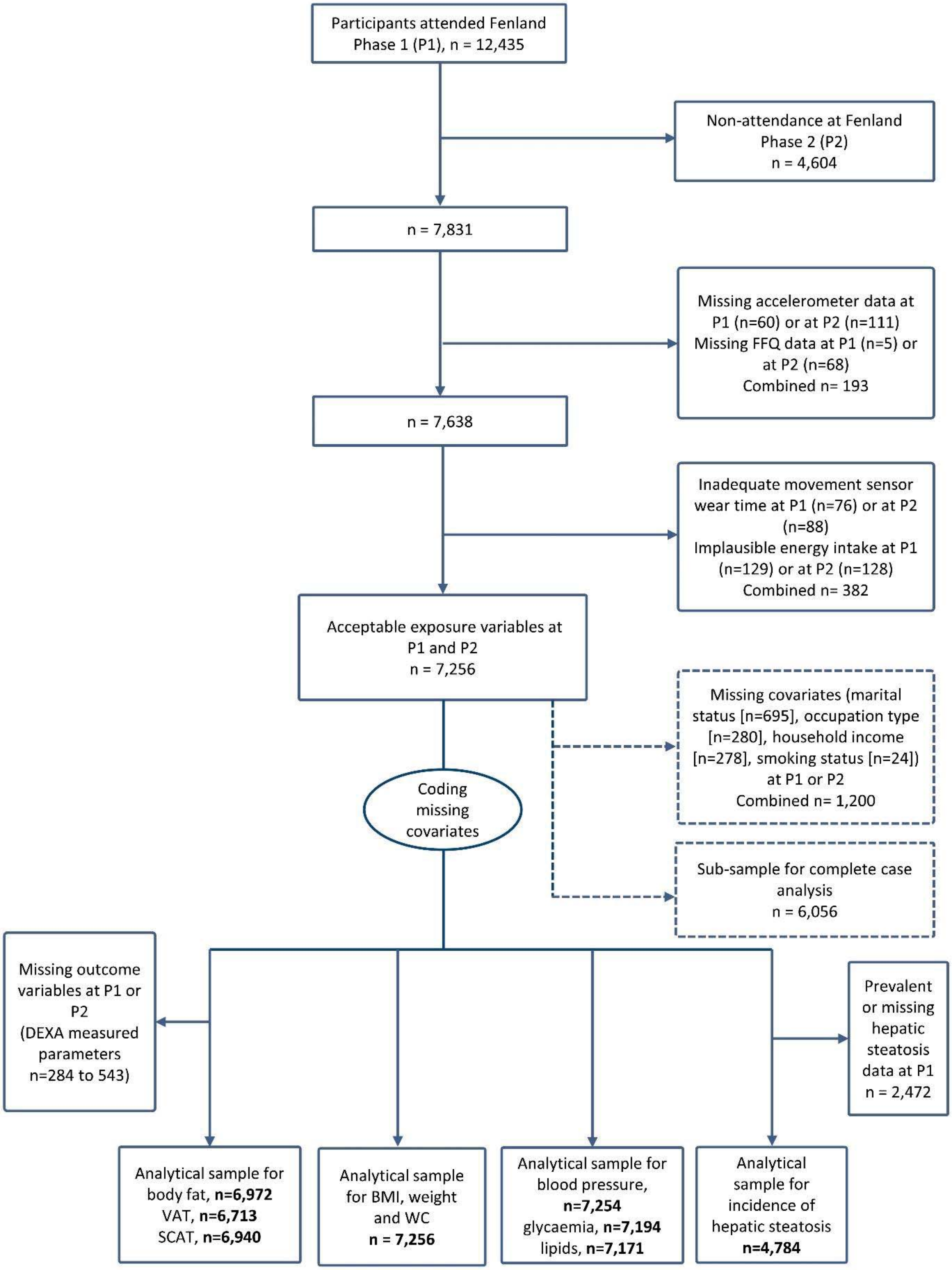

eFigure 1 - Flow diagram illustrating selection of the analytical sample in Fenland Study.

DEXA= dual energy x-ray absorptiometry; BMI=Body mass index; WC=Waist circumference; VAT=Visceral adipose tissue. SCAT= Subcutaneous adipose tissue

© 2025 Aryannezhad S et al. *JAMA Network Open*.

eTable 1 - Tests of interaction between concurrent changes over time in physical activity and diet quality in relation to adiposity markers in the Fenland Study.

	Interaction term: ΔPAEE[3] × ΔMDS[4]		
**Outcomes[1]**	β Coefficient (95%CI)[2]	P for interaction	Post-hoc statistical power[5]
ΔBMI (kg/m^2)	0.03 (-0.01 to 0.07)	0.17	31.1%
ΔWeight (kg)	0.09 (-0.03 to 0.21)	0.14	35.6%
ΔWC (cm)	0.04 (-0.09 to 0.17)	0.54	14.2%
ΔBody fat (kg)	0.08 (-0.02 to 0.18)	0.11	39.1%
ΔVAT (g)	6.8 (-2.0 to 15.6)	0.13	34.0%
ΔSCAT (g)	4.8 (-2.3 to 11.9)	0.19	28.4%
	Incidence rate ratio (95%CI)[6]		
Hepatic steatosis[7]	1.04 (0.98 to 1.11)	0.18	27.0%

1 Changes over time in markers of adiposity (value at Phase 2 – value at Phase 1)
2 Multivariable linear regression, adjusted for mutual exposures and confounders including age, sex, baseline PAEE, baseline MDS, baseline value of the outcome adiposity marker, follow-up time, test site, education, marital status, occupation type, time-updated household income, time-updated smoking status, energy intake at baseline, Δ energy intake
3 Per SD increase in ΔPAEE (equivalent to 19.0 kJ/kg/day)
4 Per SD increase in ΔMDS (equivalent to 1.27 points)
5 Post hoc power estimation was performed by bootstrap resampling (1000 times) and calculating the proportion of rejecting the null hypothesis.
6 Poisson regression, covariates in the model same as above
7 Incidence of ultrasound diagnosed hepatic steatosis at Phase 2, in those without hepatic steatosis at Phase 1.

© 2025 Aryannezhad S et al. *JAMA Network Open*.

eTable 2 - Joint associations of concurrent changes over time in physical activity and diet quality with adiposity markers in the Fenland Study.

Change in PAEE	Change in MDS	ΔBMI (kg/m²)	ΔWeight (kg)	ΔWC(cm)	ΔBody fat (kg)	ΔVAT (g)	ΔSCAT (g)
Decrease<br>-16.86 (11.7)	Decrease<br>-1.04 (0.71)	0.82<br>(0.64 to 1.01)	2.42<br>(1.88 to 2.95)	2.6<br>(2.0 to 3.2)	2.03<br>(1.6 to 2.47)	165<br>(126 to 204)	114<br>(82 to 146)
Decrease<br>-17.65 (11.29)	Stable<br>+0.33 (0.29)	0.60<br>(0.42 to 0.79)	1.83<br>(1.29 to 2.37)	1.9<br>(1.3 to 2.5)	1.5<br>(1.06 to 1.94)	116<br>(76 to 155)	83<br>(50 to 115)
Decrease<br>-16.93 (11.1)	Increase<br>+1.67 (0.73)	0.41<br>(0.22 to 0.60)	1.27<br>(0.73 to 1.82)	1.2<br>(0.6 to 1.8)	0.95<br>(0.51 to 1.39)	61<br>(21 to 101)	55<br>(22 to 87)
Stable<br>+3.04 (4.26)	Decrease<br>-1.02 (0.74)	0.10<br>(-0.08 to 0.29)	0.38<br>(-0.16 to 0.91)	0.4<br>(-0.2 to 1.0)	0.29<br>(-0.14 to 0.72)	29<br>(-10 to 67)	28<br>(-4 to 59)
Stable<br>+3.12 (4.12) 0	Stable<br>+0.33 (0.30)	0<br>(ref)	0<br>(ref)	0<br>(ref)	0<br>(ref)	0<br>(ref)	0<br>(ref)
Stable<br>+3.27 (4.18)	Increase<br>+1.66 (0.70)	-0.27<br>(-0.46 to -0.09)	-0.70<br>(-1.23 to -0.17)	-0.8<br>(-1.4 to -0.2)	-0.59<br>(-1.02 to -0.16)	-50<br>(-89 to -12)	-48<br>(-80 to -17)
Increase<br>+23.12 (10.72)	Decrease<br>-1.05 (0.66)	-0.37<br>(-0.56 to -0.18)	-1.02<br>(-1.57 to -0.48)	-0.9<br>(-1.5 to -0.3)	-1.03<br>(-1.47 to -0.59)	-75<br>(-114 to -35)	-44<br>(-77 to -12)
Increase<br>+23.22 (11.07)	Stable<br>+0.33 (0.3)	-0.56<br>(-0.74 to -0.38)	-1.60<br>(-2.13 to -1.08)	-1.6<br>(-2.2 to -1.1)	-1.51<br>(-1.94 to -1.09)	-120<br>(-158 to -83)	-82<br>(-113 to -51)
Increase<br>+23.59 (11.74)	Increase<br>+1.72 (0.75)	-0.70<br>(-0.88 to -0.52)	-2.00<br>(-2.52 to -1.47)	-2.3<br>(-2.9 to -1.7)	-1.86<br>(-2.28 to -1.44)	-149<br>(-187 to -111)	-97<br>(-128 to -66)

Results are beta coefficients and 95%CI based on multivariable linear regression, adjusted for mutual exposures and confounders including age, sex, baseline PAEE, baseline MDS, baseline value of the outcome adiposity marker, follow-up time, test site, education, marital status, occupation type, time-updated household income, time-updated smoking status, energy intake at baseline, Δ energy intake.
Numbers in each exposure category indicate mean (SD) of the ΔPAEE (kJ/kg/day) and ΔMDS (points).
Decrease, stable and increase correspond to 1st, 2nd and 3rd tertiles of change in PAEE or change in MDS.

© 2025 Aryannezhad S et al. *JAMA Network Open*.

eTable 3 - Associations of concurrent changes over time in physical activity with adiposity markers in the Fenland Study (from Phase 1 to Phase 2) per 1-SD difference in ΔPAEE in different strata of baseline sex, age, BMI, smoking, PAEE, and MDS.

	ΔBMI (kg/m^2)		ΔWeight (kg)		ΔWC (cm)		ΔBody fat (kg)		ΔVAT (g)		ΔSCAT (g)		Hepatic steatosis	
Strata (Baseline characteristics)	β Coefficient (95%CI)	P*	β Coefficient (95%CI)	P*	β Coefficient (95%CI)	P*	β Coefficient (95%CI)	P*	β Coefficient (95%CI)	P*	β Coefficient (95%CI)	P*	Incidence rate ratio (95%CI)	P*
Female	-0.65 (-0.73 to -0.57)	**<0.01**	-1.78 (-2.00 to -1.57)	**0.03**	-1.8 (-2.1 to -1.6)	0.16	-1.52 (-1.7 to -1.34)	**0.03**	-85 (-97 to -72)	**<0.01**	-102 (-116 to -88)	**<0.01**	0.78 (0.69 to 0.87)	0.37
Male	-0.45 (-0.51 to -0.40)		-1.45 (-1.63 to -1.26)		-1.6 (-1.8 to -1.4)		-1.31 (-1.46 to -1.17)		-129 (-145 to -113)		-52 (-62 to -42)		0.82 (0.73 to 0.92)	
Age < 50y	-0.56 (-0.63 to -0.49)	**<0.01**	-1.66 (-1.86 to -1.46)	**<0.01**	-1.8 (-2.0 to -1.70)	**<0.01**	-1.52 (-1.66 to -1.36)	**<0.01**	-118 (-132 to -107)	**<0.01**	-85 (-97 to -73)	**<0.01**	0.78 (0.69 to 0.87)	0.91
Age ≥ 50y	-0.49 (-0.56 to -0.42)		-1.40 (-1.59 to -1.20)		-1.5 (-1.8 to-1.3)		-1.21 (-1.37 to -1.05)		-95 (-110 to -80)		-60 (-72 to -49)		0.83 (0.74 to 0.92)	
Never smoker	-0.50 (-0.56 to -0.44)	**0.04**	-1.47 (-1.65 to -1.29)	**0.03**	-1.5 (-1.7 to -1.3)	**0.03**	-1.28 (-1.43 to -1.14)	**0.02**	-98 (-111 to -85)	0.08	-68 (-79 to -56)	**0.02**	0.85 (0.76 to 0.94)	0.29
Current or former smoker	-0.60 (-0.68 to -0.53)		-1.76 (-1.99 to -1.53)		-1.9 (-2.2 to -1.7)		-1.57 (-1.75 to -1.38)		-121 (-137 to -104)		-84 (-97 to -71)		0.74 (0.66 to 0.83)	
BMI < 25 kg/m^2	-0.37 (-0.43 to -0.31)	**<0.01**	-1.08 (-1.25 to -0.92)	**<0.01**	-1.2 (-1.4 to -1.0)	**<0.01**	-0.96 (-1.10 to -0.81)	**<0.01**	-65 (-77 to -54)	**<0.01**	-60 (-70 to -49)	0.21	0.86 (0.75 to 0.98)	0.85
BMI ≥ 25 kg/m^2	-0.60 (-0.67 to -0.53)		-1.99 (-2.20 to -1.77)		-2.0 (-2.3 to -1.8)		-1.74 (-1.91 to -1.57)		-136 (-151 to -120)		-85 (-98 to -73)		0.80 (0.73 to 0.89)	
PAEE < 50 kJ/kg/day	-0.64 (-0.73 to -0.55)	**<0.01**	-1.86 (-2.11 to -1.61)	**0.01**	-1.9 (-2.1 to -1.6)	0.10	-1.61 (-1.81 to -1.41)	**<0.01**	-120 (-137 to -103)	0.09	-87 (-102 to -73)	**0.05**	0.78 (0.68 to 0.88)	0.64
PAEE ≥ 50 kJ/kg/day	-0.47 (-0.53 to -0.42)		-1.41 (-1.57 to -1.24)		-1.6 (-1.8 to -1.4)		-1.26 (-1.40 to -1.13)		-99 (-111 to -86)		-66 (-76 to -57)		0.81 (0.73 to 0.90)	
MDS < 7.5 points	-0.53 (-0.60 to -0.46)	0.99	-1.57 (-1.78 to -1.36)	0.89	-1.7 (-1.9 to -1.5)	0.63	-1.41 (-1.58 to -1.24)	0.74	-115 (-130 to -99)	0.06	-71 (-83 to -59)	0.22	0.86 (0.78 to 0.95)	0.06
MDS ≥ 7.5 points	-0.56 (-0.62 to -0.49)		-1.61 (-1.80 to -1.42)		-1.7 (-1.9 to -1.4)		-1.40 (-1.55 to -1.24)		-101 (-115 to -88)		-80 (-92 to -68)		0.73 (0.65 to 0.83)	

Multivariable linear regression, adjusted for mutual exposures and confounders including age, sex, baseline PAEE, baseline MDS, ΔMDS, baseline value of the outcome adiposity marker, follow-up time, test site, education, marital status, occupation type, time-updated household income, time-updated smoking status, energy intake at baseline, Δ energy intake

Per SD increase in ΔPAEE is equivalent to 19.0 kJ/kg/day

* P-value for subgroup interaction; bold font indicates statistical significance

© 2025 Aryannezhad S et al. *JAMA Network Open*.

eTable 4 - Associations of concurrent changes over time in diet quality with adiposity markers in the Fenland Study (from Phase 1 to Phase 2) per 1-SD difference in ΔMDS in different strata of baseline sex, age, BMI, smoking, PAEE, and MDS.

	ΔBMI (kg/m^2)		ΔWeight (kg)		ΔWC (cm)		ΔBody fat (kg)		ΔVAT (g)		ΔSCAT (g)		Hepatic steatosis	
Strata (Baseline characteristics)	β Coefficient (95%CI)	P*	β Coefficient (95%CI)	P*	β Coefficient (95%CI)	P*	β Coefficient (95%CI)	P*	β Coefficient (95%CI)	P*	β Coefficient (95%CI)	P*	Incidence rate ratio (95%CI)	P*
Female	-0.19 (-0.27 to -0.12)	0.55	-0.52 (-0.71 to -0.32)	0.85	-0.7 (-0.9 to -0.4)	0.86	-0.45 (-0.62 to -0.29)	0.81	-32 (-43 to -21)	0.14	-33 (-45 to -20)	0.52	0.87 (0.79 to 0.96)	0.39
Male	-0.17 (-0.26 TO -0.11)		-0.54 (-0.74 to -0.31)		-0.7 (-0.9 to -0.5)		-0.5 (-0.65 to -0.35)		-61 (-78 to -44)		-23 (-33 to -13)		0.91 (0.81 to 1.02)	
Age < 50y	-0.23 (-0.30 to -0.16)	0.49	-0.66 (-0.86 to -0.46)	0.51	-0.7 (-0.9 to -0.5)	0.45	-0.57 (-0.74 to -0.41)	0.54	-52 (-66 to -38)	0.76	-35 (-47 to -23)	0.95	0.90 (0.81 to 1.00)	0.96
Age ≥ 50y	-0.15 (-0.21 to -0.08)		-0.42 (-0.61 to -0.23)		-0.7 (-0.9 to -0.5)		-0.38 (-0.53 to -0.22)		-40 (-54 to -25)		-23 (-34 to -12)		0.87 (0.78 to 0.98)	
Never smoker	-0.22 (-0.28 to -0.16)	**<0.01**	-0.63 (-0.80 to -0.45)	**0.01**	-0.7 (-0.9 to -0.5)	0.11	-0.54 (-0.68 to -0.4)	**0.03**	-46 (-59 to -33)	0.31	-34 (-45 to -24)	**0.02**	0.89 (0.80 to 0.99)	0.95
Current or former smoker	-0.13 (-0.21 to -0.05)		-0.36 (-0.59 to -0.14)		-0.6 (-0.9 to -0.4)		-0.36 (-0.55 to -0.18)		-42 (-58 to -25)		-20 (-33 to -7)		0.89 (0.80 to 1.00)	
BMI < 25 kg/m^2	-0.12 (-0.17 to -0.06)	**0.02**	-0.35 (-0.50 to -0.19)	**0.03**	-0.5 (-0.7 to-0.3)	0.14	-0.32 (-0.45 to -0.18)	**0.02**	-25 (-36 to -14)	**<0.01**	-19 (-29 to -9)	0.10	0.93 (0.82 to 1.05)	0.68
BMI ≥ 25 kg/m^2	-0.21 (-0.28 to -0.13)		-0.64 (-0.85 to -0.42)		-0.8 (-1.0 to -0.6)		-0.58 (-0.75 to -0.41)		-60 (-75 to -44)		-33 (-46 to -21)		0.89 (0.81 to 0.97)	
PAEE < 50 kJ/kg/day	-0.17 (-0.24 to -0.09)	0.68	-0.50 (-0.72 to -0.28)	0.75	-0.7 (-0.9 to -.04)	0.91	-0.42 (-0.59 to -0.24)	0.66	-48 (-63 to -33)	0.67	-23 (-36 to -10)	0.27	0.88 (0.79 to 0.99)	0.82
PAEE ≥ 50 kJ/kg/day	-0.20 (-0.26 to -0.14)		-0.55 (-0.72 to -0.37)		-0.7 (-0.9 to -0.5)		-0.52 (-0.67 to -0.37)		-44 (-57 to -30)		-32 (-42 to -21)		0.90 (0.81 to 1.00)	
MDS < 7.5 points	-0.20 (-0.27 to -0.13)	0.77	-0.55 (-0.77 to -0.34)	0.77	-0.7 (-1.0 to -0.5)	0.87	-0.50 (-0.67 to -0.33)	0.78	-48 (-66 to -32)	0.35	-31 (-44 to -19)	0.11	0.86 (0.78 to 0.96)	0.32
MDS ≥ 7.5 points	-0.17 (-0.23 to -0.11)		-0.49 (-0.67 to -0.31)		-0.6 (-0.8 to -0.4)		-0.44 (-0.58 to -0.29)		-43 (-55 to -30)		-25 (-35 to -14)		0.92 (0.83 to 1.03)	

Multivariable linear regression, adjusted for mutual exposures and confounders including age, sex, baseline PAEE, baseline MDS, ΔMDS, baseline value of the outcome adiposity marker, follow-up time, test site, education, marital status, occupation type, time-updated household income, time-updated smoking status, energy intake at baseline, Δ energy intake

Per SD increase in ΔMDS is equivalent to 1.27 points

* P-value for subgroup interaction; bold font indicates statistical significance

© 2025 Aryannezhad S et al. *JAMA Network Open*.

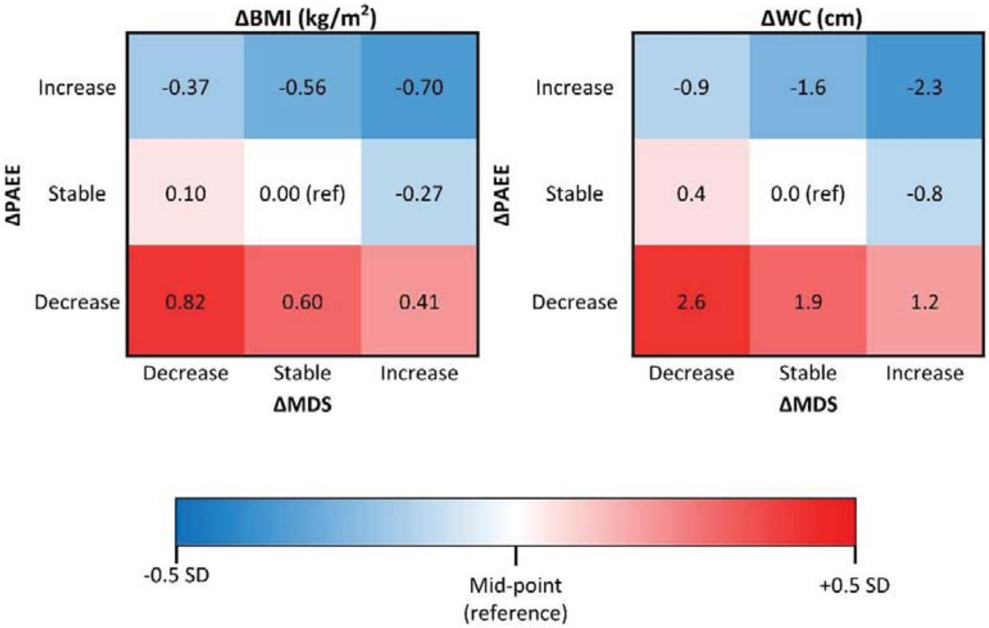

eFigure 2 - Joint associations of concurrent changes over time in physical activity and diet quality with adiposity markers in the Fenland Study.

A full blue colour indicates a reduction equivalent to 0.5 SD of the change in adiposity marker, while a full red colour indicates an increase of the same magnitude. Lighter shades reflect smaller changes in either direction.

BMI=Body mass index; WC=Waist circumference;

© 2025 Aryannezhad S et al. *JAMA Network Open*.

eTable 5 - Minimally-adjusted associations of concurrent changes over time in physical activity and diet quality with adiposity markers (from Phase 1 to Phase 2) in the Fenland Study.		
	**Exposures**	
**Outcomes**[1]	β Coefficient (95%CI)[2]	
	ΔPAEE[3]	ΔMDS[4]
ΔBMI (kg/m^2)	-0.54 (-0.59 to -0.49)	-0.19 (-0.24 to -0.15)
ΔWeight (kg)	-1.57 (-1.71 to -1.43)	-0.53 (-0.66 to -0.39)
ΔWC (cm)	-1.7 (-1.8 to -1.5)	-0.7 (-0.9 to -0.6)
ΔBody fat (kg)	-1.39 (-1.50 to -1.27)	-0.47 (-0.58 to -0.36)
ΔVAT (g)	-105 (-116 to -95)	-45 (-55 to -36)
ΔSCAT (g)	-75 (-83 to -66)	-27 (-35 to -19)
	Incidence rate ratio (95%CI)[5]	
Hepatic steatosis[6]	0.80 (0.74 to 0.86)	0.88 (0.81 to 0.94)

1 Changes over time in markers of adiposity (value at Phase 2 – value at Phase 1)
2 Multivariable linear regression, adjusted for mutual exposures and confounders including age, sex, baseline PAEE, baseline MDS, baseline value of the outcome adiposity marker, follow-up time
3 Per SD increase in ΔPAEE (equals to 19.0 kJ/kg/day)
4 Per SD increase in ΔMDS (equals to 1.27 points)
5 Poisson regression, covariates in the model same as above
6 Incidence of US diagnosed hepatic steatosis at Phase 2, in those without hepatic steatosis at Phase 1.

© 2025 Aryannezhad S et al. *JAMA Network Open*.

eTable 6 - Associations of concurrent changes over time in physical activity and diet quality with adiposity markers (from Phase 1 to Phase 2) in the Fenland Study, complete case analysis (n=6,056).

	**Exposures**	
**Outcomes[1]**	β Coefficient (95%CI)[2]	
	ΔPAEE[3]	ΔMDS[4]
ΔBMI (kg/m^2)	-0.54 (-0.59 to -0.48)	-0.17 (-0.22 to -0.11)
ΔWeight (kg)	-1.57 (-1.72 to -1.42)	-0.46 (-0.61 to -0.31)
ΔWC (cm)	-1.6 (-1.8 to -1.5)	-0.6 (-0.8 to -0.4)
ΔBody fat (kg)	-1.39 (-1.50 to – 1.27)	- 0.47 (-0.58 to -0.36)
ΔVAT (g)	-106 (-117 to -95)	-41 (-52 to -30)
ΔSCAT (g)	-75 (-84 to -66)	-24 (-33 to -16)
	Incidence rate ratio (95%CI)[5]	
Hepatic steatosis[6]	0.82 (0.75 to 0.89)	0.90 (0.83 to 0.98)

1 Changes over time in markers of adiposity (value at Phase 2 – value at Phase 1)
2 Multivariable linear regression, adjusted for mutual exposures and confounders including age, sex, baseline PAEE, baseline MDS, baseline value of the outcome adiposity marker, follow-up time, test site, education, marital status, occupation type, time-updated household income, time-updated smoking status, energy intake at baseline, Δ energy intake
3 Per SD increase in ΔPAEE (equals to 19.0 kJ/kg/day)
4 Per SD increase in ΔMDS (equals to 1.27 points)
5 Poisson regression, covariates in the model same as above
6 Incidence of US diagnosed hepatic steatosis at Phase 2, in those without hepatic steatosis at Phase 1.

© 2025 Aryannezhad S et al. *JAMA Network Open*.

eTable 7 - Associations of concurrent changes over time in physical activity and diet quality with adiposity markers (from Phase 1 to Phase 2) in the Fenland Study, additionally adjusted for seasonality.		
	**Exposures**	
**Outcomes[1]**	β Coefficient (95%CI)[2]	
	ΔPAEE[3]	ΔMDS[4]
ΔWeight (kg)	-1.59 (-1.73 to -1.45)	-0.52 (-0.66 to -0.38)
ΔBMI (kg/m^2)	-0.54 (-0.59 to -0.50)	-0.18 (-0.23 to -0.13)
ΔWC (cm)	-1.7 (-1.8 to -1.5)	-0.7 (-0.8 to -0.5)
ΔBody fat (kg)	-1.41 (-1.52 to -1.29)	-0.47 (-0.58 to -0.36)
ΔSCAT (g)	-75 (-84 to -67)	-28 (-36 to -20)
ΔVAT (g)	-108 (-118 to -98)	-45 (-55 to -35)
	Incidence rate ratio (95%CI)[5]	
Hepatic steatosis[6]	0.80 (0.74 to 0.87)	0.89 (0.82 to 0.96)

1 Changes over time in markers of adiposity (value at Phase 2 – value at Phase 1)
2 Multivariable linear regression, adjusted for mutual exposures and confounders including age, sex, baseline PAEE, baseline MDS, baseline value of the outcome adiposity marker, follow-up time, test site, education, marital status, occupation type, time-updated household income, time-updated smoking status, energy intake at baseline, Δ energy intake, seasonality of assessment (two cosinor parameters for both baseline and follow-up dates)
3 Per SD increase in ΔPAEE (equals to 19.0 kJ/kg/day)
4 Per SD increase in ΔMDS (equals to 1.27 points)
5 Poisson regression, covariates in the model same as above
6 Incidence of US diagnosed hepatic steatosis at Phase 2, in those without hepatic steatosis at Phase 1.

© 2025 Aryannezhad S et al. *JAMA Network Open*.

eTable 8 – Associations of concurrent changes over time in physical activity and diet quality with adiposity markers in the Fenland Study, additionally adjusted for family history of diabetes, menopause and hormone-replacement therapy in women.		
	**Exposures**	
**Outcomes**[1]	β Coefficient (95%CI)[2]	
	ΔPAEE[3]	ΔMDS[4]
ΔBMI (kg/m^2)	-0.54 (-0.59 to -0.49)	-0.18 (-0.23 to -0.14)
ΔWeight (kg)	-1.59 (-1.73 to -1.45)	-0.52 (-0.66 to -0.38)
ΔWC (cm)	-1.7 (-1.8 to -1.5)	-0.7 (-0.8 to -0.5)
ΔBody fat (kg)	-1.40 (-1.51 to -1.26)	-0.47 (-0.58 to -0.36)
ΔVAT (g)	-108 (-118 to -98)	-45 (-55 to -35)
ΔSCAT (g)	-75 (-83 to -67)	-28 (-36 to -20)
	Incidence rate ratio (95%CI)[5]	
Hepatic steatosis[6]	0.80 (0.74 to 0.87)	0.89 (0.82 to 0.96)

1 Changes over time in markers of adiposity (value at Phase 2 – value at Phase 1)

2 Multivariable linear regression, adjusted for mutual exposures and confounders including age, sex, baseline PAEE, baseline MDS, baseline value of the outcome adiposity marker, follow-up time, test site, education, marital status, occupation type, time-updated household income, time-updated smoking status, energy intake at baseline, Δ energy intake, family history of diabetes, menopause and hormone-replacement therapy in women

3 Per SD increase in ΔPAEE (equals to 19.0 kJ/kg/day)

4 Per SD increase in ΔMDS (equals to 1.27 points)

5 Poisson regression, covariates in the model same as above

6 Incidence of US diagnosed hepatic steatosis at Phase 2, in those without hepatic steatosis at Phase 1.

© 2025 Aryannezhad S et al. *JAMA Network Open*.

eTable 9 - Associations of concurrent changes over time in physical activity and diet quality (measured via plasma vitamin C, a biomarker of fruit and vegetable intake) with adiposity markers (from Phase 1 to Phase 2) in the Fenland Study.		
	**Exposures**	
**Outcomes[1]**	β Coefficient (95%CI)[2]	
	ΔPAEE[3]	ΔPlasma vitamin C[4]
ΔBMI (kg/m^2)	-0.55 (-0.60 to -0.50)	-0.16 (-0.21 to -0.11)
ΔWeight (kg)	-1.62 (-1.76 to -1.48)	-0.46 (-0.61 to -0.31)
ΔWC (cm)	-1.7 (-1.9 to -1.6)	-0.5 (-0.7 to-0.3)
ΔBody fat (kg)	-1.40 (-1.54 to -1.31)	-0.37 (-0.49 to -0.24)
ΔVAT (g)	-111 (-121 to -100)	-36 (-47 to -25)
ΔSCAT (g)	-77 (-85 to -69)	-16 (-25 to -7)
	Incidence rate ratio (95%CI)[5]	
Hepatic steatosis[6]	0.79 (0.72 to 0.85)	0.95 (0.88 to 1.04)

1 Changes over time in markers of adiposity (value at Phase 2 – value at Phase 1)
2 Multivariable linear regression, adjusted for mutual exposures and confounders including age, sex, baseline PAEE, baseline plasma vitamin C, baseline value of the outcome adiposity marker, follow-up time, test site, education, marital status, occupation type, time-updated household income, time-updated smoking status, energy intake at baseline, Δ energy intake,
3 Per SD increase in ΔPAEE (equals to 19.0 kJ/kg/day)
4 Per SD increase in ΔPlasma vitamin C (equals to 60.0 µmol/L)
5 Poisson regression, covariates in the model same as above
6 Incidence of US diagnosed hepatic steatosis at Phase 2, in those without hepatic steatosis at Phase 1.

© 2025 Aryannezhad S et al. *JAMA Network Open*.

eTable 10 - Cross-sectional associations of physical activity and diet quality with adiposity markers in the Fenland Study at Phase 1.		
	**Exposures**	
**Outcomes**[1]	β Coefficient (95%CI)[2]	
	PAEE[3]	MDS[4]
BMI (kg/m^2)	-1.02 (-1.12 to -0.91)	-0.53 (-0.63 to -0.42)
Weight (kg)	-2.82 (-3.15 to -2.49)	-1.41 (-1.74 to -1.07)
WC (cm)	-2.7 (-3.0 to -2.5)	-1.5 (-0.17 to -1.2)
Body fat (kg)	-2.57 (-2.78 to -2.36)	-1.18 (-1.40 to -0.97)
VAT (g)	-162 (-178 to -146)	-87 (-104 to -71)
SCAT (g)	-155 (-171 to -139)	-62 (-78 to -46)
	Prevalence rate ratio (95%CI)[5]	
Hepatic steatosis[6]	0.77 (0.72 to 0.82)	0.89 (0.84 to 0.94)

1 Markers of adiposity measured via DEXA and abdominal US at Phase 1
2 Multivariable linear regression, adjusted for mutual exposures and confounders including age, sex, test site, education, household income, occupation type, smoking status, marital status, energy intake
3 Per SD increase in PAEE measured at Phase 1 (equivalent to 22.0 kJ/kg/day)
4 Per SD increase in MDS measured at Phase 1 (equivalent to 1.47 points)
5 Poisson regression, covariates in the model same as above
6 Prevalent ultrasound diagnosed hepatic steatosis at Phase 1

© 2025 Aryannezhad S et al. *JAMA Network Open*.

eTable 11 - Longitudinal associations of baseline physical activity and diet quality (Phase 1) with subsequent changes in adiposity markers (from Phase 1 to Phase 2) in the Fenland Study.

	**Exposures**	
**Outcomes**[1]	β Coefficient (95%CI)[2]	
	Baseline PAEE[3]	Baseline MDS[4]
ΔBMI (kg/m^2)	-0.19 (-0.24 to -0.14)	-0.15 (-0.20 to -0.09)
ΔWeight (kg)	-0.51 (-0.66 to -0.36)	-0.40 (-0.55 to -0.24)
ΔWC (cm)	-0.7 (-0.9 to -0.6)	-0.7 (-0.8 to -0.5)
ΔBody fat (kg)	-0.53 (-0.65 to -0.40)	-0.35 (-0.47 to -0.22)
ΔVAT (g)	-43 (-54 to -32)	-32 (-43 to -21)
ΔSCAT (g)	-25 (-34 to -16)	-23 (-32 to -14)
	Incidence rate ratio (95%CI)[5]	
Hepatic steatosis[6]	0.82 (0.75 to 0.89)	0.79 (0.73 to 0.86)

1 Changes over time in markers of adiposity (value at Phase 2 – value at Phase 1)
2 Multivariable linear regression, adjusted for mutual exposures and confounders including age, sex, marital status, ΔPAEE, ΔMDS, baseline value of the outcome adiposity marker, follow-up time, test site, education, occupation type, time-updated household income, time-updated smoking status, energy intake at baseline, Δ energy intake
3 Per SD increase in PAEE measured at Phase 1 (equals to 22.0 kJ/kg/day)
4 Per SD increase in MDS measured at Phase 1 (equals to 1.47 points)
5 Poisson regression, covariates in the model same as above
6 Incidence of US diagnosed hepatic steatosis at Phase 2, in those without hepatic steatosis at Phase 1.

© 2025 Aryannezhad S et al. *JAMA Network Open*.

eTable 12 - Associations of concurrent changes over time in physical activity and diet quality with adiposity markers relative to body weight in the Fenland Study (from Phase 1 to Phase 2).		
	**Exposures**	
**Outcomes[1]**	β Coefficient (95%CI)[2]	
	ΔPAEE[3]	ΔMDS[4]
Δbody fat as percentage of body weight (%)	-1.06 (-1.15 to -0.97)	-0.36 (-0.45 to -0.28)
ΔVAT as percentage of body weight (%)	-0.10 (-0.11 to -0.09)	-0.04 (-0.05 to -0.03)
ΔSCAT as percentage of body weight (%)	-0.06 (-0.07 to -0.05)	-0.02 (-0.03 to -0.01)

1 Changes over time in markers of adiposity (value at Phase 2 – value at Phase 1). Markers of adiposity are expressed as change in proportion of total body weight.
2 Multivariable linear regression, adjusted for mutual exposures and confounders including age, sex, baseline PAEE, baseline MDS, baseline value of the outcome adiposity marker, follow-up time, test site, education, marital status, occupation type, time-updated household income, time-updated smoking status, energy intake at baseline, Δ energy intake
3 Per SD increase in ΔPAEE (equals to 19.0 kJ/kg/day)
4 Per SD increase in ΔMDS (equals to 1.27 points)

© 2025 Aryannezhad S et al. *JAMA Network Open*.

eTable 13 – Associations of concurrent changes over time in physical activity and diet quality with adiposity markers in the Fenland Study, expressed in SD units.

	Exposures	
**Outcomes in SD units[1]**	β Coefficient (95%CI)[2]	
	ΔPAEE[3]	ΔMDS[4]
ΔBMI	-0.27 (-0.3 to -0.25)	-0.09 (-0.12 to -0.07)
ΔWeight	-0.28 (-0.3 to -0.25)	-0.09 (-0.11 to -0.07)
ΔWC	-0.26 (-0.29 to -0.24)	-0.11 (-0.13 to -0.08)
ΔBody fat	-0.31 (-0.33 to -0.28)	-0.10 (-0.13 to -0.08)
ΔVAT	-0.27 (-0.3 to -0.24)	-0.11 (-0.14 to -0.09)
ΔSCAT	-0.22 (-0.24 to -0.19)	-0.07 (-0.09 to -0.04)

1 Changes over time in markers of adiposity (value at Phase 2 – value at Phase 1) expressed in SD units. Equivalent SD values for each outcome are presented in main Table 1.

2 Multivariable linear regression, adjusted for mutual exposures and confounders including age, sex, baseline PAEE, baseline MDS, baseline value of the outcome adiposity marker, follow-up time, test site, education, marital status, occupation type, time-updated household income, time-updated smoking status, energy intake at baseline, Δ energy intake

3 Per SD increase in ΔPAEE (equals to 19.0 kJ/kg/day)

4 Per SD increase in ΔMDS (equals to 1.27 points)

© 2025 Aryannezhad S et al. *JAMA Network Open*.

eTable 14 - Longitudinal associations of baseline physical activity and diet quality (at phase 1) and changes over time in them (from Phase 1 to Phase 2) with incidence of overweight or obesity (from Phase 1 to Phase 2) in the Fenland Study.

	**Exposures**			
**Outcomes**	Incidence rate ratio (95%CI)[1]			
	Baseline PAEE[2]	Baseline MDS[3]	ΔPAEE[4]	ΔMDS[5]
Overweight or obesity[6]	0.82 (0.74 to 0.90)	0.87 (0.79 to 0.96)	0.69 (0.63 to 0.76)	0.90 (0.83 to 0.99)

1 Poisson regression, adjusted for mutual exposures and confounders including age, sex, baseline value of the outcome adiposity marker, follow-up time, test site, education, time-updated household income, occupation type, time-updated smoking status, marital status, energy intake at baseline, Δ energy intake
2 Per SD increase in PAEE measured at Phase 1 (equals to 21.6 kJ/kg/day)
3 Per SD increase in MDS measured at Phase 1 (equals to 1.46 points)
4 Per SD increase in ΔPAEE (equals to 19.0 kJ/kg/day)
6 Per SD increase in ΔMDS (equals to 1.27 points)
6 Incidence of overweight or obesity at Phase 2, in those with normal BMI (<25kg/m^2) at Phase 1 (n=3,067).

© 2025 Aryannezhad S et al. *JAMA Network Open*.

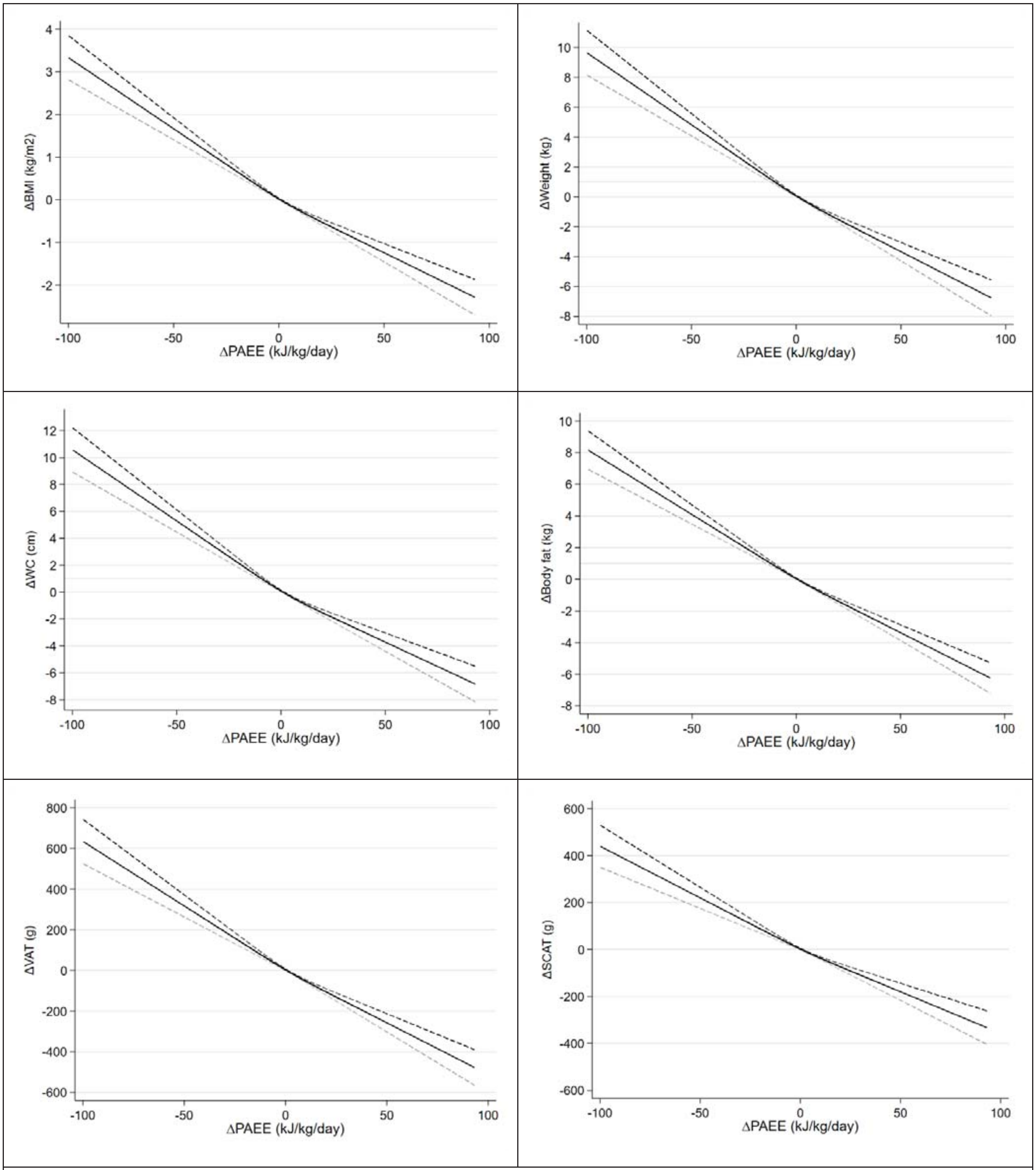

eFigure 3 – Dose-response relationship between changes over time in PA (ΔPAEE) and changes in adiposity markers using restricted cubic spline regression.
Confounder adjusted multivariable model was used for this analysis (see methods)
The 95% CI is depicted with dashed lines
ΔPAEE= changes over time in physical activity energy expenditure

© 2025 Aryannezhad S et al. *JAMA Network Open*.

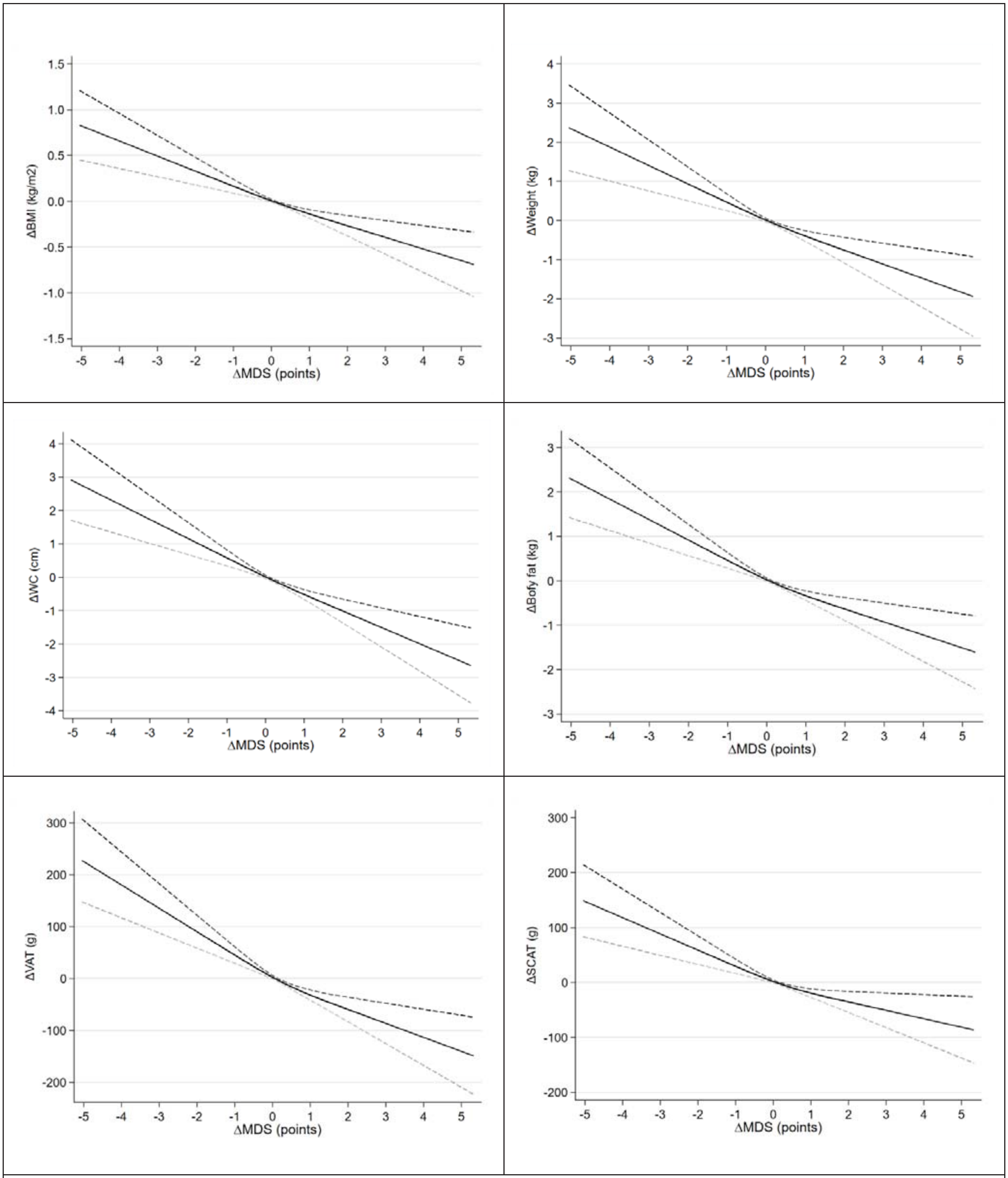

eFigure 4 – Dose-response relationship between changes over time in diet quality (ΔMDS) and changes in adiposity markers using restricted cubic spline regression.
Confounder adjusted multivariable model was used for this analysis (see methods)
The 95% CI is depicted with dashed lines
MDS=Mediterranean diet score, ΔMDS= changes over time in MDS

© 2025 Aryannezhad S et al. *JAMA Network Open*.

eReferences

1. Bingham SA, Gill C, Welch A, Cassidy A, Runswick SA, Oakes S, et al. Validation of dietary assessment methods in the UK arm of EPIC using weighed records, and 24-hour urinary nitrogen and potassium and serum vitamin C and carotenoids as biomarkers. Int J Epidemiol. 1997;26 Suppl 1:S137-51.
2. Bach-Faig A, Berry EM, Lairon D, Reguant J, Trichopoulou A, Dernini S, et al. Mediterranean diet pyramid today. Science and cultural updates. Public Health Nutr. 2011;14(12a):2274-84.
3. Khalatbari-Soltani S, Imamura F, Brage S, De Lucia Rolfe E, Griffin SJ, Wareham NJ, et al. The association between adherence to the Mediterranean diet and hepatic steatosis: cross-sectional analysis of two independent studies, the UK Fenland Study and the Swiss CoLaus Study. BMC Med. 2019;17(1):19.
4. Tong TY, Wareham NJ, Khaw KT, Imamura F, Forouhi NG. Prospective association of the Mediterranean diet with cardiovascular disease incidence and mortality and its population impact in a non-Mediterranean population: the EPIC-Norfolk study. BMC Med. 2016;14(1):135.
5. Brage S, Brage N, Franks PW, Ekelund U, Wareham NJ. Reliability and validity of the combined heart rate and movement sensor Actiheart. Eur J Clin Nutr. 2005;59(4):561-70.
6. Brage S, Ekelund U, Brage N, Hennings MA, Froberg K, Franks PW, et al. Hierarchy of individual calibration levels for heart rate and accelerometry to measure physical activity. J Appl Physiol (1985). 2007;103(2):682-92.
7. Stegle O, Fallert SV, MacKay DJ, Brage S. Gaussian process robust regression for noisy heart rate data. IEEE Trans Biomed Eng. 2008;55(9):2143-51.
8. Brage S, Brage N, Franks PW, Ekelund U, Wong MY, Andersen LB, et al. Branched equation modeling of simultaneous accelerometry and heart rate monitoring improves estimate of directly measured physical activity energy expenditure. J Appl Physiol (1985). 2004;96(1):343-51.
9. Brage S, Westgate K, Franks PW, Stegle O, Wright A, Ekelund U, et al. Estimation of Free-Living Energy Expenditure by Heart Rate and Movement Sensing: A Doubly-Labelled Water Study. PLoS One. 2015;10(9):e0137206.
10. Lindsay T, Wijndaele K, Westgate K, Dempsey P, Strain T, De Lucia Rolfe E, et al. Joint associations between objectively measured physical activity volume and intensity with body fatness: the Fenland study. Int J Obes (Lond). 2022;46(1):169-77.
11. De Lucia Rolfe E, Brage S, Sleigh A, Finucane F, Griffin SJ, Wareham NJ, et al. Validity of ultrasonography to assess hepatic steatosis compared to magnetic resonance spectroscopy as a criterion method in older adults. PLoS One. 2018;13(11):e0207923.

© 2025 Aryannezhad S et al. *JAMA Network Open*.
