# Supplementary material for: Concurrent Changes in Diet Quality and Physical Activity and Association With Adiposity in Adults
Source: JAMA Netw Open. 2025 Nov 21;8(11):e2545232. doi: 10.1001/jamanetworkopen.2025.45232 (PMC12639474; doi:10.1001/jamanetworkopen.2025.45232)
Supplement: Supplement 2. — Data Sharing Statement [file jamanetwopen-e2545232-s002.pdf]

Data Sharing Statement

Aryannezhad. Concurrent Changes in Diet Quality and Physical Activity and Association With Adiposity in Adults. JAMA Netw Open. Published online November 21, 2025. doi:10.1001/jamanetworkopen.2025.45232

Data
Data available: No

Additional Information
Explanation for why data not available: The datasets generated and analysed during the current study are available at request via the MRC Epidemiology website (http://www.mrc-epid.cam.ac.uk/research/data-sharing/).
